# Label‐Free Near‐Infrared Plasmonic Sensing Technique for DNA Detection at Ultralow Concentrations

**DOI:** 10.1002/advs.202000763

**Published:** 2020-10-19

**Authors:** Shimeng Chen, Chuan Liu, Yun Liu, Qiang Liu, Mengdi Lu, Sheng Bi, Zhenguo Jing, Qingxu Yu, Wei Peng

**Affiliations:** ^1^ School of Optoelectronic Engineering and Instrumentation Science Dalian University of Technology Dalian 116024 China; ^2^ State Key Laboratory of Structural Analysis for Industrial Equipment Dalian University of Technology Dalian 116024 China; ^3^ School of Physics Dalian University of Technology Dalian 116024 China; ^4^ Key Laboratory for Precision and Non‐traditional Machining Technology of the Ministry of Education Dalian University of Technology Dalian 116024 China

**Keywords:** biosensing, nanostructure, plasmonic sensors, signal enhancement, ultrasensitivity

## Abstract

Biomolecular detection at a low concentration is usually the most important criterion for biological measurement and early stage disease diagnosis. In this paper, a highly sensitive nanoplasmonic biosensing approach is demonstrated by achieving near‐infrared plasmonic excitation on a continuous gold‐coated nanotriangular array. Near‐infrared incident light at a small incident angle excites surface plasmon resonance with much higher spectral sensitivity compared with traditional configuration, due to its greater interactive volume and the stronger electric field intensity. By introducing sharp nanotriangular metallic tips, intense localization of plasmonic near‐fields is realized to enhance the molecular perception ability on sensing surface. This approach with an enhanced sensitivity (42103.8 nm per RIU) and a high figure of merit (367.812) achieves a direct assay of ssDNA at nanomolar level, which is a further step in label‐free ultrasensitive sensing technique. Considerable improvement is recorded in the detection limit of ssDNA as 1.2 × 10^−18^ m based on the coupling effect between nanotriangles and gold nanoparticles. This work combines high bulk‐ and surface‐sensitivities, providing a simple way toward label‐free ultralow‐concentration biomolecular detection.

## Graphical abstract

A highly sensitive nanoplasmonic biosensing approach is reported by achieving near‐infrared plasmonic excitation on a continuous gold‐coated nanotriangular array. This sensor possesses an enhanced sensitivity of 42 103.8 nm per RIU and a high figure of merit of 367.812. This technical approach is used to detect single‐stranded DNA hybridization adsorption events by using gold nanoparticles and achieves an estimated detection limit of 1.2 × 10^−18^ m.

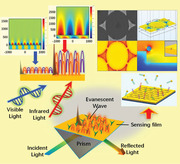

## 1 Introduction

Surface plasmons confined to a metallic/dielectric interface are widely known to be sensitive to dielectric refractive index (RI) changes within the decay length of an evanescent field.^[^ ^1^ ^]^ Based on this optical characteristic, plasmonic sensors have attracted huge attentions because of their advantages like high sensitivity, real‐time response, and label‐free.^[^ ^2^ ^]^ As a kind of alternatives to traditional immunoassays, it's ideal for detecting surface bioaffinity adsorption of biological and chemical analytes.^[^ ^3^, ^4^, ^5^ ^]^ However, plasmonic sensing technique has been facing the technical challenge in detecting small analytes at low concentrations below the picomole level. The limits of detction (LODs) of most plasmonic biosensors are insufficient to detect trace amounts of small molecules for early stage disease diagnosis.^[^ ^6^ ^]^ To achieve the lower LOD of conventional plasmonic sensors, drastic sensitivity enhancement is crucial to sensing applications.

For plasmonic sensors relying on RI detection to monitor biological binding behaviors, there are two important RI sensitivity metrics: bulk‐sensitivity and surface‐sensitivity.^[^ ^7^ ^]^ The bulk‐sensitivity is defined as the variation in the characteristics of reflected light (wavelength, angle, phase, etc.) when per RI unit is changed in bulk medium within the evanescent field.^[^ ^8^, ^9^ ^]^ Many approaches have been proved to improve bulk‐sensitivity of surface plasmons. Long‐range SPR sensors were reported to achieve a slightly higher sensitivity and a smaller full width at half maximum (FWHM),^[^ ^10^, ^11^, ^12^ ^]^ which is due to the source energy concentration and the propagation length increase. Another effective enhancement approach was reported to add thin dielectric materials with a high RI onto the sensing surface. And many dielectric layers, such as ZrO_2_, Si, ITO, and TeO_2_,^[^ ^13^, ^14^, ^15^, ^16^ ^]^ were introduced to induce the larger evanescent field. However, a significant drawback of the above two approaches is that sensing structures limit the biomolecular modification and their application in biosensing fields. A sensitivity‐enhancement approach was presented by modifying the prism RI.^[^ ^17^, ^18^ ^]^ The sensitivity increases with the decreasing RI contrast between the prism and the analyte, but the adverse effects are the larger FWHM and the lower dynamic detecting range. Phase‐sensitive SPR sensor was extensively studied based on different interference methods,^[^ ^19^, ^20^, ^21^ ^]^ because it possesses high resolution and small LOD. Nevertheless, it's extremely limited due to high requirement for stability and small detection range. In addition, infrared SPR sensors based on angle‐modulation were proposed to achieve propagation length and depth,^[^ ^22^, ^23^ ^]^ which is more appropriate for researching cells. But angular sensitivity of the infrared sensor is lower than that of visible range. Conversely, the wavelength‐modulated SPR sensor has deeper decay depth and higher sensitivity in infrared region,^[^ ^24^, ^25^ ^]^ and even this character has rarely been utilized in the biosensing field. In addition to the bulk‐sensitivity, the surface‐sensitivity is also important for evaluating the performance of biosensors, because any thickness or density changes of the biomolecular adsorption layer on the surface can induce signal variation of the incident light. With the development of increasingly sophisticated nanoscale manipulation, nanoplasmonic sensors have been paid wide attention in biosensing fields due to its remarkable surface‐sensitivity and optimized transmission performance. A wide variety of complex nanostructures and their assemblies were designed, such as nanoslit,^[^ ^26^, ^27^ ^]^ nanohole^[^ ^28^, ^29^ ^]^ and nanodisk.^[^ ^30^, ^31^ ^]^ Metallic nanostructures can obtain strong enhancement of the local field and large confinement, which can achieve the high sensitivity and the low LOD. However, strong local field enhancement comes at the expense of a reduced bulk‐sensitivity and a decreased decay depth. What is more, expensive fabrication processes also limit their applications to some biological species. Besides, plasmonic sensors modified by nanomaterials (graphene,^[^ ^32^, ^33^ ^]^ nanorod,^[^ ^34^, ^35^, ^36^ ^]^ porous anodic alumina,^[^ ^37^, ^38^ ^]^) on sensing surface have been reported to improve surface‐sensitivity and widely applied for small molecule sensing. Enhanced local field arises from the charge transfer and substantial overlap of excited field between the nanomaterial and the sensing film. Due to their complicated chemical synthesis or nanofabrication, most nanomaterials are not suitable for broad applications.

To overcome the measurement bottlenecks associated with plasmonic sensors for RI and biological sensing, it's urgent to develop novel plasmonic sensors with simple technical approaches to obtain high sensitivity and low LOD. In this paper, a novel near‐infrared (NIR) plasmonic sensor for ultrasensitive biological sensing was designed based on a continuous gold‐coated nanotriangular (NT) array sensing structure, so as to achieve high bulk‐ and surface‐sensitivity simultaneously.The stronger plasmonic field and the higher sensitivity of 42 103.8 nm per RIU can be achieved in NIR excitation waveband. We further enhanced the detection level for small molecules by using plasmonic coupling between gold nanoparticles (GNPs) and extremely strong local electric near‐fields around the NT array. This technical approach is used to detect single‐stranded DNA (ssDNA) hybridization adsorption events. *Rpo*B is related to rifampicin drug resistance^[^ ^39^ ^]^ about tuberculosis treatment. Mutations in *Rpo*B that confer resistance to rifamycin do so by altering residues of the rifamycin binding site on RNA polymerase, thereby reducing rifamycin binding affinity for rifamycins. We detected *RpoB* with an estimated LOD of 1.2 × 10^−18^ m, which is more than 2–4 orders of magnitude better than that of most reported plasmonic sensors.

## 2 Results and Discussion

### 2.1 NIR Surface Plasmon Resonance (SPR) Sensing

**Figure 1:**
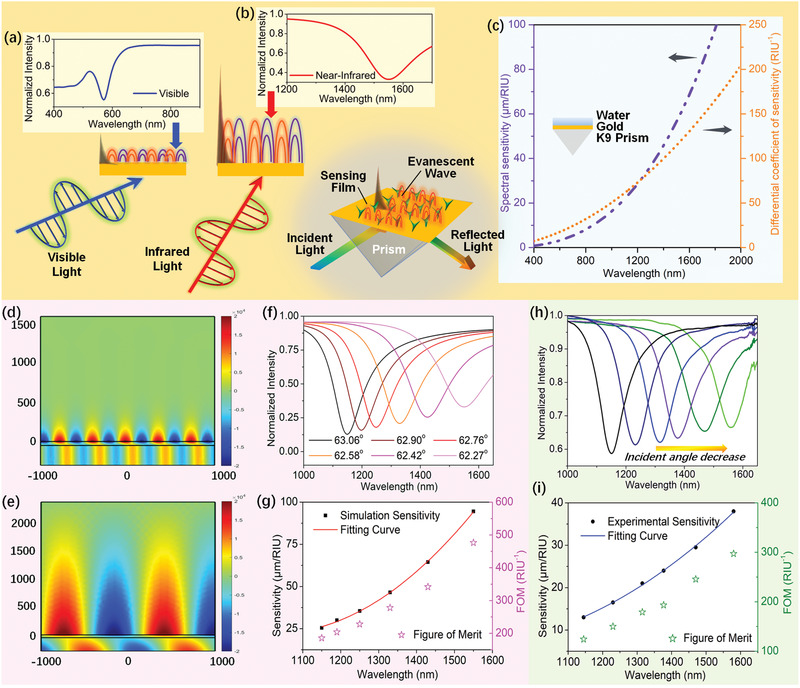
Study design and schematic diagram of a NIR plasmonic biosensor based on Kretschmann geometry with K9 prism and 50 nm gold film. Simulated reflection spectrum in visible band with an incident angle of 85° a) and NIR band with an incident angle of 62° b). c) MATLAB simulation results of the spectral sensitivity and its differential coefficient with the resonance wavelength between 400 nm and 2000 nm. Electric field distribution (*x* component of the field E_x_) appearing in visible band (570 nm) and d) NIR band (1550 nm). e). COMSOL simulation results of spectra f) and sensitivity per FOM g) of NIR plasmonic sensor with different incident angles. Experimental results of spectra h) and sensitivity per FOM i) of NIR plasmonic sensor with different incident angles.

Unlike the traditional SPR sensor, excited by visible light at a large incident angle (**Figure** **1**a), we presented an NIR plasmonic sensor and demonstrated the effectiveness of the sensing element operating in NIR band (Figure 1b) in this section. The prism‐coupled SPR sensor with spectral interrogation is based on the Kretschmann geometry of attenuated total reflection method.^[^ ^40^ ^]^ The resonant excitation condition of the surface plasmon wave can be shown that *Re* (*k*) = *k*_0_ *n_p_*sin*α*,^[^ ^41^ ^]^ where *k* is the propagation constant at the interface between metal and dielectric, *n_p_* is the RI of the prism, *α* is the incident angle in the prism, *k*_0_ =  *ω*/*c* is the free space wavenumber of optical wave, and *ω* and *c* are the angular frequency and the light velocity. We can obtain *k*  = *k*_0_ [*ε*_*m*_ *ε*_*d*_/(*ε*_*m*_ + *ε*_*d*_)]^1/2^ of Kretschmann's geometry based on solving Maxwell's equations with resonance boundary conditions, where *ε*_*d*_ and *ε*_*m*_ are the permittivities of dielectric and metal. We used Drude model to describe the dispersion characteristics of gold^[^ ^42^ ^]^

(1) $$\begin{array}{c} \varepsilon_{m}\;\left(\omega \right)=\;1-\frac{\Omega_{p}^{2}}{\omega^{2}-i\gamma \omega }\;\left(\;\Omega_{p}^{2}=\;f\omega_{p}^{2}\right) \end{array}$$

where *ω*_*p*_ is the plasma frequency and Ω_*p*_ =  2*πc*/*λ*_*p*_ is the plasma frequency associated with intraband transitions with oscillator strength *f* and damping constant *γ*. Parameter values of Drude model are shown in Table S1 in the Supporting Information. *ε*_*m*_ depends strongly on the wavelength and can be expressed as a function of resonant wavelength (*ε*_*m*_ (*λ*) = *ε*_*mr*_  + *iε*_*mi*_). As for gold |*ε*_*mr*_| ≫ *ε*_*mi*_,^[^ ^43^ ^]^ the imaginary part of *ε*_*m*_ can be neglected, and the real parts of *k* and *ε*_*m*_(*λ*) can be obtained that

(2) $$\begin{array}{c} Re\left(k\right)\approx k_{0}\sqrt{\frac{\varepsilon_{mr}\left(\lambda \right)\varepsilon_{d}}{\varepsilon_{mr}\left(\lambda \right)+\varepsilon_{d}}} \end{array}$$

(3) $$\begin{array}{c} \varepsilon_{mr}\;\left(\lambda \right)=\;1-\frac{f\omega_{p}^{2}\lambda^{2}}{4\pi^{2}c^{2}+\gamma^{2}\lambda^{2}} \end{array}$$

Based on Equation (2), the resonance condition for the SPR sensor with spectral interrogation is

(4) $$\begin{array}{c} \frac{\varepsilon_{mr}\left(\lambda \right)n_{d}^{2}}{\varepsilon_{mr}\left(\lambda \right)+n_{d}^{2}}=n_{p}^{2}\;sin^{2}\left(\alpha \right) \end{array}$$

where, *α* remains unchanged, the resonant wavelength (*λ*) varies by d*λ* due to the RI of the dielectric (*n_d_*) changes by *dn_d_*. For the prism made of commonly used glass, the material dispersion is very small (|*dn_d_*/d*λ*| < 10^−4^|). We can neglect the dispersion effect for the prism in this paper. By differentiating Equation (4) in *λ* and *n_d_*, we can obtain that

(5) $$\begin{array}{c} D_{\varepsilon }n_{d}^{3}d\lambda \;=\;-2\varepsilon_{mr}^{2}\left(\lambda \right)dn_{d} \end{array}$$

where *D*_*ε*_ =  *dε*_*mr*_(*λ*)/*dλ* is the differential coefficient of *ε*_*mr*_ with respect to *λ*. The spectral sensitivity is defined as the wavelength variation when per RI unit is changed in bulk medium (d*λ*/*dn_p_*) and can be obtained by Equation (5)

(6) $$\begin{array}{c} S\;\left(\lambda \right)=\;-\frac{2\varepsilon_{mr}^{2}\left(\lambda \right)}{n_{d}^{3}D_{\varepsilon }} \end{array}$$

Due to $\gamma^{2}\ll f\omega_{p}^{2}$, the sensitivity can be approximated as

(7) $$\begin{array}{c} S\left(\lambda \right)\approx \frac{1}{n_{d}^{3}}\left(\frac{\lambda_{p}^{2}}{\lambda }-2\lambda +\frac{\lambda^{3}}{\lambda_{p}^{2}}\right) \end{array}$$

The spectral sensitivity as a function of wavelength is shown as purple line in Figure 1c using MATLAB software. It can be clearly seen that the sensitivity increases monotonously with wavelength in the simulated wavelength range (400–2000 nm). Besides, the sensitivity has also been found to be inversely proportional to $n_{d}^{3}$, so the higher sensitivity can be expected if there is a smaller RI of the dielectric medium. For example, SPR sensor with the same resonance wavelength should be more sensitive to the gas sample than the liquid sample.

In order to further research the SPR field distribution characteristics of different incident angles, the electric field distributions were researched. TM waves are directed along 5° and 28° from *x*‐axis at wavelengths of 570ands1550 nm. And the thickness of gold film is set as 50 nm (ThepropertiesofNIR‐SPR withdifferent thicknesses are illustratedas shown in Figure S1in the Supporting Information). The *x* component of field E_x_ and intensity|*E*|for visible andNIR bandsare simulated by COMSOL softwareasshown in Figure 1d,e and FigureS2 in the Supporting Information.The gold‐water interface exhibits a prominent evanescent field in the surrounding medium. The intensity and electric field in NIR band is much greater than that in the visible band. The typical decay length in water at visible wavelength is ≈200 nm. Nevertheless, the field at NIR wavelength extends and enhances substantially, and produces an evanescent wave penetrating over 2000 nm. This substantial increase is attributed to change in the RI of gold. The confinement dimensions of the decay length inside the metal is $\delta_{d}=\left(\lambda /2\pi \right)\;\sqrt{\left(\varepsilon_{d}+\varepsilon_{mr}\right)/\left(-\varepsilon_{d}^{2}\right)}$. And the real part of the gold RI decreases significantly at longer wavelengths, which demonstrates longer decay length of the electric fields associated with surface plasmons at NIR band relative to visible band (as shown in Figure S3 in the Supporting Information). Sensitivity improvement is associated with the enhancement of the electromagnetic field overlap integral describing the interactive energy within the sample.^[^ ^44^ ^]^ Since the SPR is accompanied by an enhanced evanescent field in the metal/analyte interface region, the sensor sensitivity for a perturbation in the analyte is determined by the field distribution in this region. The wave equation can be expressed as ∇  ×  ∇  ×  E  = k^2^  · *ε* · E, where E, k are the electric field and the wavevector. Assuming a RI alteration is added to the dielectric sample, which caused a permittivitychange $\left(\delta \varepsilon \;=\varepsilon_{d}\;-\varepsilon_{d}^{{}^{\prime }}\right)$in the interactive volume, and the variatitons inthe electric field $\left(\delta \;\vec{E}=\vec{E}\;-{\vec{E}}^{{}^{\prime }}\right)$and the wavevector (*δ*k  =  k − k′). Introducing the altered values into wave equation, multiplying by ${\vec{E}}^{*}$ and integrating over the entire volume, leads to

(8) $$\begin{array}{c} \left(k^{2}-k^{{}^{\prime }2}\right)\int_{V}{\vec{E}}^{{}^{\prime }}\cdot \varepsilon \cdot {\vec{E}}^{*}\mathrm{dr}\;=k^{{}^{\prime }2}\;\int_{V_{\mathrm{int}}}{\vec{E}}^{*}\cdot \delta \varepsilon \cdot {\vec{E}}^{{}^{\prime }}\mathrm{dr} \end{array}$$

Using first‐order perturbation theory in k, we can get

(9) $$\begin{array}{c} \delta k\approx \frac{k}{2}\frac{\int_{V_{\mathrm{int}}}\delta \varepsilon {\vec{E}}^{*}{\vec{E}}^{{}^{\prime }}\mathrm{dr}}{\int_{V}\varepsilon {\vec{E}}^{*}\vec{E}\mathrm{dr}} \end{array}$$

For the wavelength‐modulation SPR sensor, *δk*is the correlative variation due to the permittivity change (*δε*)and expressed by resonant wavelength shift. (*δk*/*δε*)can represent thesensitivity of wavelength‐modulation SPR sensor, which is proportionalto the overlap integral in the numerator of Equation (9).And the overlap integral in turn is proportional to theinteractivevolume *V_int_*and the electric field *E*′. Increasing the interactive volume *V*_int_ (including decay depth and propagation length of surface plasmon) and the field intensity in the sensing region can improve sensitivity. Therefore, the SPR sensor can excite the deeper and stronger field at NIR wavelength, thus allowing it to achieve higher sensitivity within the sensing region.

The reflection spectra with different incident angles are calculated in Figure 1f to investigate the spectral performanceinNIR band.And the spectral responseswith different RIs are shown in Figure S4 in the Supporting Information. The results clearly show that resonance wavelength redshifts when incident angle decreases, and the same RI change can cause larger wavelength shift as the resonance wavelength redshifts. Figure 1g shows the simulation results for variations in RI sensitivity at different resonance wavelengths. The results also suggest that surface plasmons excited at long wavelengths are more sensitive to environmental RI variations than those excited at short wavelengths, which reaches a very good agreement between the simulation results of MATLAB and COMSOL. In addition, it can be found that although FWHM increases with increasing resonance wavelength, the figure of merit (FOM) still increases with increasing resonance wavelength. Furthermore, the experimental reflection spectra with different incident anglesweretested asshown in Figure 1h. Higher sensitivity and larger FOM at longer wavelengths has been confirmed experimentally in Figure 1i, which are in good agreement with simulation results.

### 2.2 Surface‐Sensitivity Enhancement Using the Gold‐Coated NT Array

**Figure 2:**
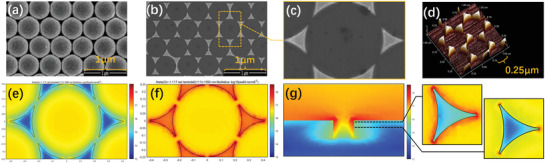
Schematic representation of the gold‐coated NT sensing array: a) SEM image of fabricated deposition masks produced with 500 nm PS spheres. SEM images of the NT array ofmulti‐period b) and single‐period c). d)AFM images of the NT array. Electric field distributions numerically evaluated over a single period in the X‐Y plane for *Z* = 50 nm e) and *Z* = 100 nm f) and in the cross section of the gap g); inset of g): Electric field distribution of a single NT plate in the *X*–*Y* plane for *Z* = 75 nm (Left) and Z = 99 nm (Right).

Here, we applied a novel nanostructured metallic surfaces with enhanced sensing capability instead of the usual flat gold film. A gold NT array with uniform segment sphere voids was distributed on the bottom, and a gold film was deposited on a nanoarray to form a continuous metallic nanostructure. **Figure** **2**a,b shows typical scanning electron microscopy (SEM) images of deposition masks with polystyrene (PS) spheres and the NT array. The SEM image of one period structure with a gap between adjacent NT planes is shown in Figure 2c. Then we researched the nanostructure‐sized feature of the NT array by atomic force microscopy (AFM), as shown in Figure 2d. These micrographs show that the spherical voids are uniform and smooth after removing the PS sphere template. In order to investigate the properties and characteristics of the NT array, we built a simulation model structure of one complete period, as shown in Figure S5 in the Supporting Information. Due to the precision limitation of the evaporation coating technique, metal films cannot be coated in the tangential position between PS spheres. The gap was set to 50 nm, and the TM wave was directed along 26° at incident light wavelength of 1550 nm. As shown in Figure 2e,f, the central areas of the NT terraces exhibit larger field intensity distributions than the gold film. The introduction of the NT array resulted in local enhancement, which led to higher surface‐sensitivity toward molecular binding in the enhanced local fields. On the other hand, the NT array shows the effect of a significantly enhanced plasmonic near‐fields, which focuses on the sharp corners and edges. The field distribution in the cross section of the gap (Figure 2g) shows that the plasmonic near‐fields are the most intense at the vertices of the NT array, followed by the edges. Besides, we also investigated the properties of sensing element with different dimensions, and we simulated the characteristic of spectrum and electric field (see Figure S6, Supporting Information). The resonance wavelength is red‐shifted with the diameter decreasing while spectral shapes are similar.The near‐field distributions of NT array show similar property that there are strong enhancements around edge and vertex. Because the local field strength in the vicinity of a sharp tip can be enhanced by several orders of magnitude compared with the incident light,^[^ ^45^ ^]^ metallic nanostructures with sharp corners or edges are especially favorable for the detection of specific interactions between biologically relevant species. In addition, the large surface area‐to‐volume ratio of this sensing chip allows highly effective surface‐based biomolecular mass sensing.

**Figure 3:**
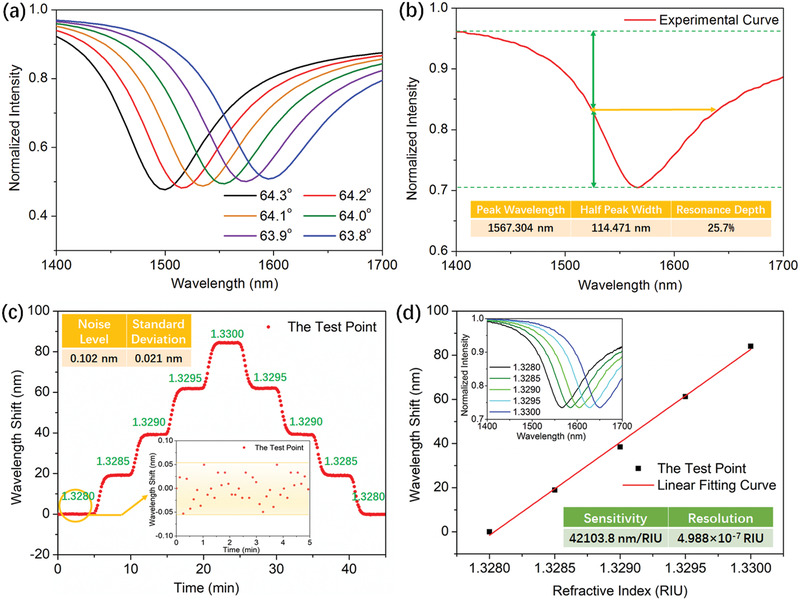
Demonstration of a NIR plasmonic sensor based on an NT array: a) Theoretical reflection spectrum with different incident angles. b) Normalized reflection spectrum of the experimental results. c) Time response for solutions with different RIs; inset: Stability test. d) Calibration curve of wavelength variation versus RIs; inset: Reflection spectra with RI changes.

Simulation results for spectra of NIR plasmonic sensor based on continuous gold‐coated NT array at different incident angles are shown in **Figure** **3**a using COMSOL software.By comparingflat gold film and gold‐coated NT array, we can find that the spectral shapes are similar and the resonance wavelengths of the same incident angles are red‐shifted by introducing the NT array (Figure S7a,b, Supporting Information).In addition, we also simulated the characteristic of the electric field distribution as shown in Figure S7c,d in the Supporting Information. The electric field distributions of a large region in dielectric layer are similar for two cases, and the introduction of NT array only causes enhancement of the near field around the array.Next, we experiemently demonstratedits sensing potential,the incident angle was 64° in all of the following experiments. Figure 3b shows the reflection spectrum at the resonance wavelength of 1567.304 nm, and the half peak width and the resonance depth are 114.471 nm and 25.7%, respectively. We analyzed the RI sensitivity by using sodium chloride solutions at different concentrations. The results, summarized in Figure 3c, clearly show that the resonance peak redshifts when RI increases and the response can return to the initial value when deionized water is pumped. In our work, resonant wavelength is identified by valley‐detection based on spline interpolation algorithm and the experimental error is less than ±28.5 pm (see Figure S8in the Supporting Information). The sensing system has high stability, and the noise level of the sensing system is 0.102 nm. The standard deviation of the baseline is 0.021 nm, which is helpful for the sensing system to obtain a high resolution and a low LOD. The sensitivity is calculated as 42 103.8 nm per RIU in Figure 3d and exceeds the sensitivity of traditional plasmonic sensors in visible band by one order of magnitude.^[^ ^46^ ^]^ It can be found that the NIR plasmonic sensorwith NT array also possesses ultrahigh sensitivity. It verifies the above simulation result for bulk‐sensitivity. The numerical calculation is related to the bulk‐sensitivity, which is based on a large sensing region in the dielectric layer. Thus, it can be applied to the sensor with NT array structure to analyze the characteristic of bulk‐sensitivity. Moreover, the resolution of the sensing system was calculated to be 4.988 × 10^−7^ RIU, which achieved a resolving power comparable to typical phase‐modulated SPR sensor.^[^ ^47^ ^]^ In addition, the FOM of our sensing system reached a value of 367.812. This parameter exceeds those of most reported plasmonic sensors.

### 2.3 Biosensing Application of NIR Nanoplasmonic Sensor

**Figure 4:**
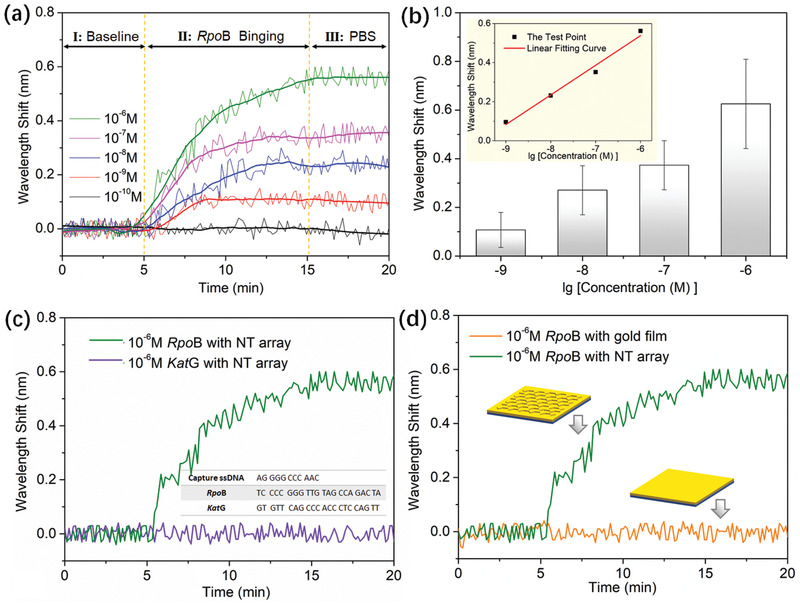
ssDNA hybridization analyses by using a NIR plasmonic sensor based on an NT array: a) Real‐time responses for detection of *RpoB* at concentrations of 10^−10^–10^−6^ m. b) Mean and standard deviation of five samples for duplicate detection of three times; inset: The relationship between wavelength response and sample concentration ([Wavelength Response] = 1.352 + 0.138[Concentration]). c) Contrast responses for the detection of *RpoB* and *Kat*G at concentrations of 10^−6^ m. d) Contrast responses for detection of *RpoB* at concentrations of 10^−6^ m corresponding to an NT array sensor and a gold film sensor.

Furthermore, the high‐sensitivity performance of our sensor can be selected to test small biomolecule, giving access to a low concentration detection that is important for many tasks in disease diagnosis. To examine the biosensing feasibility of our sensor, we detected the sequences of the *RpoB* and *Kat*G genes, which are related to drug resistances of rifampicin and isoniazid for treating tuberculosis infection.^[^ ^48^ ^]^ The NT array chip functionalized with capture ssDNA was used for real‐time detection of *RpoB* binding. The real‐time wavelength shift was measured during ssDNA hybridization process. **Figure** **4**a shows the real‐time responses of *RpoB* solution at concentrations of 10^−10^–10^−6^ m. The wavelength red shifts due to *RpoB* binding to capture ssDNA in the sensing region. When the concentration decreased to 10^−10^ m, there was no response because the concentration was less than the LOD of the sensing system. The mean values and standard deviations were calculated by repeated experiments as shown in Figure 4b. An approximately linear function was observed between the response and the *RpoB* concentration in the inset. Next, we detected the *Kat*G solution to successfully verify the specificity of the sensing system as shown in Figure 4c. In particular, NIR plasmonic sensor based on the NT array can be used as a bioanalysis platform to directly assay the ssDNA solution at low concentrations, while NIR plasmonic sensor without the NT array cannot measure the *RpoB* hybridization process (Figure 4d). The nanomolar detection range can be reached without any chemical or biological amplification due to the effect of the NT array on the sensing surface, which can increase the local field intensity and improve the surface‐sensitivity. Surface‐sensitivity is related to near‐field distribution,^[^ ^31^, ^34^, ^49^, ^50^ ^]^ so the characteristic of the near‐field distribution corresponding to flat gold film and gold‐coated NT array were simulated as shown in Figure S9 in the Supporting Information. Compared with flat gold film, ssDNA is located the stronger near‐field around edge and vertex, which can result in high surface‐sensitivity of ssDNA specific binding. In addition, the increased surface areas provided by the NT array also improved the amount of molecules involved in hybridization and enhanced the surface‐sensitivity. The LOD of the NT array‐based plasmonic sensor was calculated to be 0.456 × 10^−9^ m.

**Figure 5:**
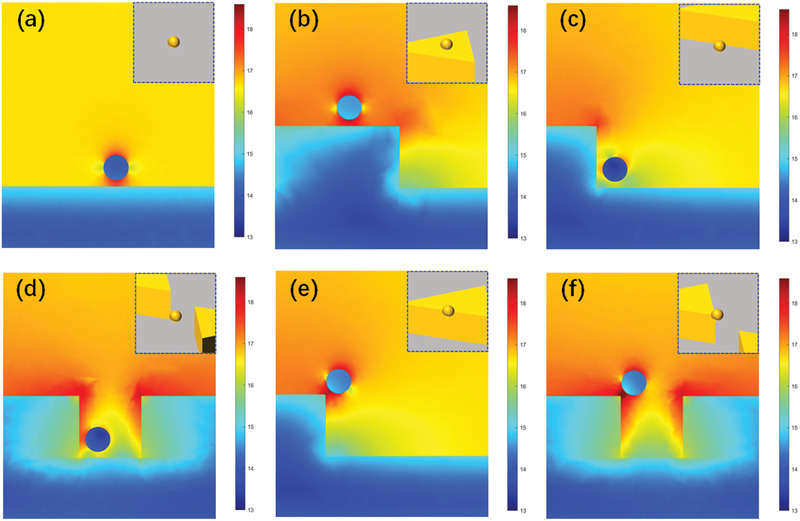
Simulations of the electric field distributions for the coupling between a single GNP at different locations of an NT array: The GNP is placed near a) the single gold film, b) the terrace of the NT plate, c) the side face of the NT plate, d) the lateral edge of the NT plate, e) the edge of the terrace of the NT plate, and f) the vertex of the NT plate.

GNPs were chosen as amplification tags to further trigger secondary signal amplification, which is due to the large permittivity and surface mass loading, as well as the coupling effect between sensing surface and GNPs. The electric field distribution of GNPs coupled with a gold‐coated NT array structure was studied in **Figure** **5**. We simulated the coupling plasmonic near‐fields and the effect of the attachment location of the GNPs attached to the NT plate. A single GNP was placed at typical locations as shown in the inset of Figure 5, including plane, edge and vertex, respectively. The GNP was placed ≈5 nm away from the sensing surface because GNPs are linked to the sensing array by DNA. The field distribution shows the strong dipolar mode excited between the NT plate and the GNP. The GNP exhibited an asymmetric field distribution. And the electric field is concentrated in the gap between the GNP and the sensing array. This result indicates that there was a strong plasmonic coupling of the NT array with the GNP. The different enhancement effects of the localized electric field depended on the location of the GNP on the sensing array (Figure 5). In order to better compare the electric field changes when GNPs are introduced to carry out coupling with the NT plates, we studied the electric field distributions in both directions: i) X‐Y plane in Figure S10 in the Supporting Information and ii) the different planes perpendicular to the sensing surface in Figure S11 in the Supporting Information. When the GNP is located above the NT terrace, the plasmonic coupling is stronger than that of the single gold film, which is related to the plasmonic near‐field distribution of the NT array. Of all cases considered, the GNP located at the vertex resulted in the strongest coupling, followed by the GNP located at the edge, while the GNP located on the plane resulted in the weakest coupling. This can be explained as a heterodimers effect of plasmonic nanoparticles, which consists of GNP and NT plate. Heterodimers can prove new coupling modes that are not possessed withhomodimers. And many core‐satellite nanostructures have been researched on their plasmonic coupling effect.^[^ ^51^, ^52^, ^53^ ^]^ Plasmoniccoupling of heterodimers is strongly dependent on the mutual location,^[^ ^54^ ^]^ and the coupling effect is strongly dependent on the site of GNPs attachment in our structure.There are two reasons that lead to the occurrence of the strongest electric coupling when the GNP is attached to the vertex of the NT plate: i)The observed location dependence is due to the spatial variations in the polarizability of NT plate. When sensing structure is excited by incident light, NT plate is the most polarizable at the vertices, followed by the edges, which are served by simulation of electric field distributionin different cross sections as shown in the inset of Figure 2g. ii) There is strong coupling when the excitation is polarized along the axis of NT plate and GNP, which can lead to alignment of dipolar plasmon oscillation.It is indicative that an attractive interaction is induced by the dipolar plasmon oscillation of both. Thus, the strongest electric coupling occurs when the GNP is attached to the vertex of the NT plate, whereas other locations possess weaker coupling. In our experiment, a large ratio of ssDNA/GNP (200:1) was used to achieve great uniformity of the coverage.Ultimately, their overall coupling effect in all location cases led to the enhancement of the detection signal and surface‐sensitivity. In addition to coupling effect, the large permittivity and surface mass loading of GNPs also enhanced the surface‐sensitivity of plasmonic sensor.

**Figure 6:**
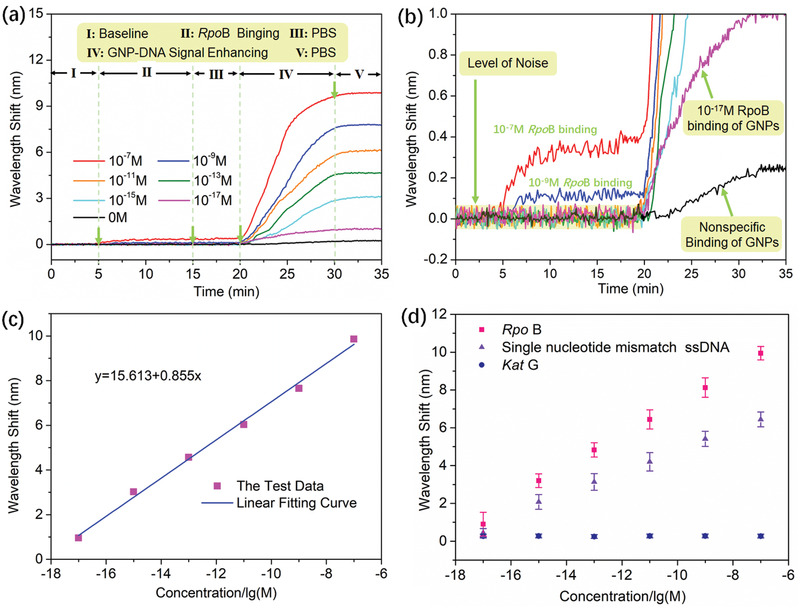
DNA hybridization analyses by using GNP amplification tags: a) Wavelength shift at *RpoB* concentrations ranging from 10^−17^ to 10^−7^ m. b) Partial enlargement of (a). c) Relationship between the measured wavelength shift and the sample concentration. d) Wavelength resonance during the hybridization of GNP‐DNA and *RpoB*/single nucleotide mismatch ssDNA/*Kat*G; the error bars in the diagram indicate the relative error of the variability among samples of the same concentration in three replicated tests.

We detected *RpoB* gene by sandwich amplification strategy. Enhancement effect of GNPs with common dimensions (diameters of 20 and 40 nm) were analyzed by simulation and experiment as shown in Figure S12 in the Supporting Information. GNPs with a diameter of 40 nm, which can obtain the greater enhancement, were used as amplification tags in ourexperiments. A weak response can be obtained when *RpoB* solution was injected into the flow cell. The sensing signal immediately increased when pumping GNP‐DNA solution. Next, *RpoB* solutions with different concentrations from 10^−17^ to 10^−7^ m were tested. And the real‐time response curves are shown in **Figure** **6**a. The binding processes were fast, ≈10 min. The responses proportionally increased with *RpoB* concentration, indicating that more GNP‐DNAs hybridized in the same time. The sandwich assay with GNPs achieved the detection of *RpoB* levels as low as 10^−17^ m. To evaluate the surface adhesion influence of GNPs and the sensing surface, we tested the response of GNP‐DNA solution in the absence of *RpoB*. The response curve (black line) displays a weak signal in Figure 6b, which is attributed to the presence of the nonspecific adsorption of GNPs to the sensing surface. Experiment result of *Rpo*B solution at concentrations below 10^−17^ m are shown in Figure S13 in the Supporting Information.It can be found that the response differences between samples with and without *Rpo*B are less than 0.06 (triple standard deviation).The response signal of *Rpo*B solution at concentrations below 10^−18^ m is difficult to be accurately distinguished from the signal of surface adhesion influence. As shown in Figure 6c, our sensor possesses a good linearity (R^2^ = 0.995) between wavelength shift and *RpoB* concentration in the range of 10^−17^–10^−9^ m. Then, the LOD of our sensing system was estimated to be ≈1.2 × 10^−18^ m, which is far below the value of a direct assay and more than 2–4 orders of magnitude better than that of most reported plasmonic sensors. Compared with bulk‐sensitivity, surface‐sensitivity is more important for a biosensor. Wediscussed the surface‐sensitivity of NIR plamsonic sensor based on NT array in terms of RI.The surface RI sensitivity was estimated to exceed 4.742 × 10^7^ nm per RIU when all molecules pumped into flow cell are bound to the sensing surface. But there are some molecules that arenot bound to the sensing surface in theexperiment, the real surface RI sensitivity is more than 4.742 × 10^7^ nm per RIU.It far exceeds the bulk RI sensitivity dueto a combination of the enhancement effect of the NT array and the coupling effect of GNPs.

As the negative control meaurements, single nucleotide mismatch ssDNA and noncomplementary ssDNA (*Kat*G) at concentrations of 10^−7^ m under the same condition were analyzed as shown in Figure S14 in the Supporting Information. Due to its weaker specific binding, single nucleotide mismatch ssDNA should result in a smaller hybridization response than the complementary target ssDNA (*RpoB*) at the same concentration. Therefore, we can use the response amplitude of the sensing signal to distinguish mismatch ssDNA from fully complementary target ssDNA. Except for the response amplitude of the signal, we can also use the response speed to discriminate mismatch ssDNA from complementary ssDNA. Comparing the wavelength response of complementary ssDNA and mismatch ssDNA (the orange dotted lines in Figure S14 in the Supporting Information), we can find the single nucleotide mismatch ssDNA achieved smaller response speed than complementary one. By observing the response amplitude and speed from the binding curve, we can distinguish the complementary ssDNA and the single nucleotide mismatch ssDNA. The single nucleotide mismatch ssDNA might be bound with the capture ssDNA and GNP‐DNA under some conditions illustrated in Supporting Information. For noncomplementary ssDNA, the response for *Kat*G was much lower than those of *RpoB* and mismatch ssDNA. The negligible response was quite similar to that of PBS alone because nonspecific binding occurred between the *Kat*G and the capture ssDNA. In order to verify the reproducibility of ourexperimental results, the sensing chip was regenerated in high concentration urea (8 m) by ultrasonication in high temperature (80°) for 60 min. First, we tested the *Rpo*B at a concentration of 10^−7^ m in three replicated tests, as shown in Figure S15a in the Supporting Information. We can find that our sensor possesses good reproducibility and the wavelength shifts of three tests are similar.Then, each measurement (*RpoB*/single nucleotide mismatch ssDNA/*Kat*G) at a given concentration was repeated thrice. Wavelength resonance during the hybridization of GNP‐DNA and *Rpo*B/single nucleotide mismatch ssDNA/*Kat*G is shown in Figure 6d. Our sensor can distinguish the complementary ssDNA, single nucleotide mismatch ssDNA and noncomplementary ssDNA in different concentrations by comparing theresponse amplitudes and speeds from the binding curve. Besides, the error bars in the diagram indicate the relative errors of the variability among samples at the same concentration in three replicated tests.The standarddeviations of all reproducibility tests were less than 0.629 (Table S2, Supporting Information). Our sensor possesses the great repeatability and the ultralow LOD, which allows us to follow the real‐time interactive process of trace amounts of small molecules and obtain kinetic constants.

### 2.4 Comparison

To further verify the enhancement efficiency of NIR plasmonic sensor based on gold‐coated NT array, a comparison between our work and recently reported works based on various nanoplasmonic sensors for small molecule detection was performed (**Table** **1**). The biomolecular sensitivities and specificities of these enhancement strategies surpass those of most plasmonic biosensors, which demonstrate the high suitability of nanostructures and nanomaterials for the detection of small molecules and low‐concentration samples. The LOD of the sensor presented in this paper can reach attomolar level, which is better than the ones of these plasmonic sensors. In addition, in comparison to plasmonic biosensors, this NIR biosensor possesses excellent performance (sensitivity of 42 103.8 nm per RIU and resolution of 4.988 × 10^−7^ RIU) and a simple fabrication process, which are superior to those of other plasmonic sensors.

**Table 1:** Comparison of the nanoplasmonic sensing performance for small molecule detection

Enhancement Strategy	Analyte	Molecular Weight	LOD
Nanorod Metamaterials^[^ ^34^ ^]^	Biotin	244 kDa	300 × 10^−9^ m
Gold‐coated and Porous Anodic Alumina Layer^[^ ^37^ ^]^	ssDNA	6.9 kDa	10 × 10^−12^ m
3D Nanogap Arrays^[^ ^55^ ^]^	t‐DNAs	7.2 kDa	0.66 × 10^−12^ m
Graphene Oxide‐coated Gold Film^[^ ^32^ ^]^	csDNA	6.9 kDa	10 10^−15^ m
Triangular Silver Nanoprism^[^ ^56^ ^]^	ssDNA	7.3 kDa	6 10^−15^ m
Graphene‐coated Gold Film^[^ ^33^ ^]^	ssDNA	4.9 kDa	500 × 10^−18^ m
Antimonene Nanomaterial^[^ ^57^ ^]^	miRNA	6.8 kDa	10 × 10^−18^ m
Chiral Nanorod Assemblies^[^ ^58^ ^]^	ssDNA	6.9 kDa	3.7 × 10^−18^ m
Gold‐coated NT array of this work	*RpoB*	7.3 kDa	1.2 × 10^−18^ m

## 3 Conclusion

In this paper, we presented a NIR plasmonic biosensor based on a gold‐coated NT array for ultralow bioligical concentration detection. Simulation result validated that NIR wave can excite surface plasmon resenance with larger interactive volume (inculding decay depth and propagation length) and greater electric field intensity, which explains that longer excitation wavelength can result in greatly improved bulk‐sensitivity. Compared to traditional SPR sensor, the sensitivity of our work is increased by an order of magnitude and the FOM is also much better than most reported SPR sensors. The continuous NT array nanostructure was further introduced to provide enhanced surface‐sensitivity owing to field enhancement around the sharp tips of the NT array. The effectiveness and high‐sensitivity of the sensor was confirmed through direct ssDNA detection of nanomolar concentration level without any amplification method. Besides, we used GNPs coupled to nanostructures to achieve quantitative detection of ssDNA hybridization in the large dynamic range and provide an ultralow LOD.

In summary, the label‐free biological sensing platform was certified to possess a bulk‐sensitivity of 42 103.8 nm per RIU and an LOD of 1.2 × 10^−18^ m. Because of simple scheme, real‐time response and label‐free performance, this sensing system is promising for constructing high‐performance biomedical sensing platform. This technique can be further investigated for monitoring of dynamical cell processes due to its great decay length.

## 4 Experimental Section

##### Reagents

A solution of PS spheres was purchased from Tianjin Dae Technology Co. 6‐Mercapto‐1‐hexanol (MCH) was purchased from Shanghai TCI Chemical Industry Co. Tris‐(2‐carboxyethyl)‐phosphine hydrochloride (TCEP) and ultrapure water (18.2 MΩ cm) were purchased from Shanghai Sheng Gong Co. Ltd. PBS (pH 7.4) and other chemicals were purchased from Sigma‐Aldrich; the DNA wash buffer contained 10  × 10^−3^ m sodium citrate and 2% PEG, and the hybrid buffer contained 2% PEG and 10 × 10^−3^ m PBS. All DNA single strand sequences were synthesized by Shanghai Sheng Gong Co. Ltd.; The DNA sequences are listed in Table S3.

##### Experimental Setup

We used Kretschmann geometry to excitate surface plasmon and employed an isosceles right‐angled triangle prism of K9 glass with the sensing substrate (Figure S16, Supporting Information). The sensing structure was fabricated on a glass sheet (20 mm × 20 mm × 1 mm), which was mounted on the top of a prism with RI‐matching fluid and was fixed in a home‐made flow cell (*Φ*15 mm × 2 mm). The sensing configuration was illuminated by a light spot (*Φ*5 mm) from a halogen lamp (HL‐2000‐FHSA, covering the wavelength range of 200–2500 nm). Collimated broadband light was generated through a series of lenses via a multimode fiber and reflected on the top side of the prism with a sensing substrate. We collected the reflected beam by using an optical spectrum analyzer (AQ6370, via an FC/PC coupler) via multimode fiber and objective lens. The sensing nanostructure was brought into contact with a flow cell. The sample solutions were introduced to the cell by using a peristatic pump (BT‐100 2J).

##### Self‐Assembly Process of PS Spheres

The nanostructured surfaces were produced by vacuum thermal evaporation coating through a hexagonal close‐packed (HCP) colloidal sphere monolayer template.PS spheres self‐assembled into a large‐area HCP pattern on the glass substrate by the Langmuir‐Blodgett (LB) process.^[^ ^59^, ^60^, ^61^, ^62^ ^]^ LB method has been used in the preparation of nanostructure mask.^[^ ^61^, ^62^, ^63^, ^64^, ^65^, ^66^, ^67^, ^68^ ^]^ The self‐assembly process of PS spheres is as follows: First, the glass substrate (2 cm × 2 cm) was cleaned with acetone, ethanol, and deionized water in an ultrasonic bath, and soaked in piranha solution (H_2_O_2_:H_2_SO4 = 3:7, volume ratio) for 30 min to increase the hydrophilicity of the surface. And it was subsequently rinsed with deionized water and finally dried with nitrogen stream. At the same time, PS sphere suspensions were sonicated for 10 min to break up the aggregates. Then, the PS spheres were sufficiently dispersed in ethanol/water solution (volume ratio 1:1) by ultrasonic dispersion at a concentration of 2.5 wt. % for 20 min. Above all, the sodium dodecyl sulfate solution (1%, 20 µL) was added to water in a petri dish. PS sphere solution (10 µL) was dropped onto a flat glass slide tilted at the edge of the petri dish by the liquid gun. PS spheres quickly spread on the water surface and self‐assembled HCP monolayer PS spheres on the water surface, which was left for 30 min to reach a better thermodynamic equilibrium and dense HCP pattern. And then, the monolayer PS array was lifted onto the glass slides. As the solvent slowly evaporated and glass substrate dried in air, the PS spheres self‐assembled into a large‐area HCP pattern.

##### NT Array Sensing Structure Fabrication

A 50 nm gold film was deposited onto the substrate with HCP monolarer PS spheres by thermal evaporation. PS spheres were removed in 95% ethanol solution by ultrasonication for 5 min after deposition. Since the deposited gold reached the glass substrate only through the gaps in the close‐packed film, it resulted in a honeycomb NT array. Finally, we deposited a 50 nm gold film on the nanostructured films. By this preparation process (as shown in Figure S17 in the Supporting Information), the sensing nanostructured film containing a regular hexagonal NT array of uniform segment sphere voids was coated on the glass substrate.

##### NT Array Sensing Surface Preparation

Thiolated capture ssDNA was incubated in TCEP solution (twice the concentration of the capture ssDNA) at room temperature for 1 h to reduce the disulfide bonds of ssDNA.^[^ ^69^ ^]^ TCEP can reduce most stable water‐soluble alkyl disulfides. The NT array sensing surface was immersed in the mixed liquor of capture ssDNA (1 × 10^−6^ m final concentration) and MCH (1 × 10^−6^ m final concentration) in KH_2_PO_4_ buffer solutions (1 m final concentration, pH 3.8) for 2 h and then transferred to an aqueous solution of 1 × 10^−3^ m MCH for 1 h.^[^ ^70^ ^]^ The high concentration of MCH can remove the physically adsorbed capture ssDNA and block the unreacted surface, which can reduce nonspecific binding. The NT array sensing surface was rinsed thoroughly with ultrapure water and dried.

##### Preparation of GNP Solutions

GNP solutions were prepared by using the citrate reduction method.^[^ ^71^ ^]^ HAuCl_4_·3H_2_O (10 mg) was dissolved in 100 mL of ultrapure water and heated until boiling. Then, 10 mg mL^−1^ sodium citrate solution was added, the volume of which can adjust the diameter of GNPs. The solution was boiled with stirring for 15 min before cooling to room temperature. Using this method, we produced GNP solutions with diameters of 20 and 40 nm. As shown in Figure S18 in the Supporting Information, GNPs with different sizes were observed in the TEM images with different scales.

##### Functionalization of GNPs with ssDNA

GNPs were functionalized with the thiolated probe ssDNA according to previous reports.^[^ ^72^ ^]^ First, 990 µL of GNP solution and 10 µL of 100 × 10^−6^ m probe ssDNA were mixed for 30 min at room temperature. Then, 0.24 mL of 0.2% poly(ethylene glycol) (PEG) solution was added for 30 min.^[^ ^73^ ^]^ The mixed solution was salt‐aged by adding 0.125 mL of 0.3 m sodium chloride every 10 min a total of 5 times, which was followed by incubation for 12 h at room temperature. To remove excess ssDNA, we used wash buffer to wash the GNP‐DNA solution by centrifugation. Then, the GNP‐DNA conjugates were resuspended in hybrid buffer and stored at 4 °C until use. As depicted in Figure S19 in the Supporting Information, the spectrum of GNPs showed aresonance wavelength redshift of ≈4 nmand a color change of the solution following hybridization,which resulted from the formation of DNA around GNPs. This is due to the formation of nanoparticle aggregates. The plasmon band as characteristic of GNPs is very sensitive to interparticle distance as well as aggregate size. The observation of absorbance can provide compelling evidence to verify the synthesis of GNP/DNA complex.

##### Calculation of the Spectrum and Electric Field

To numerically investigate the characteristic spectra and field distribution of our designed sensor, we used the MATLAB and COMSOL software. The simulation result was based on the wavelength‐modulating Kretschmann configuration, and the simulation parameters were consistent with the experimental conditions. The RI of gold used for the simulation was taken from the Johnson data. The field was depicted in the form of lgE^2^ on a color scale.

## Conflict of Interest

The authors declare no conflict of interest.

## Supporting information

Supporting Information
