# Supplementary material for: Label‐Free Near‐Infrared Plasmonic Sensing Technique for DNA Detection at Ultralow Concentrations
Source: Adv Sci (Weinh). 2020 Oct 19;7(23):2000763. doi: 10.1002/advs.202000763 (PMC7709993; doi:10.1002/advs.202000763)
Supplement: Supplementary file 1 — Supporting Information [file ADVS-7-2000763-s001.pdf]

© 2020 Wiley-VCH GmbH

ADVANCED SCIENCE Open Access

Supporting Information

for *Adv. Sci.,* DOI: 10.1002/advs.202000763

Label-Free Near-Infrared Plasmonic Sensing Technique for DNA Detection at Ultralow Concentrations

Shimeng Chen, Chuan Liu, Yun Liu*, Qiang Liu, Mengdi Lu, Sheng Bi, Zhenguo Jing, Qingxu Yu, Wei Peng*

Supporting Information

Label-Free Near-Infrared Plasmonic Sensing Technique for DNA Detection at Ultralow Concentrations

Shimeng Chen, Chuan Liu, Yun Liu, Qiang Liu, Mengdi Lu, Sheng Bi, Zhenguo Jing, Qingxu Yu, Wei Peng**

Mrs. S. Chen, Prof. Q. Yu
School of Optoelectronic Engineering and Instrumentation Science, Dalian University of Technology, Dalian 116024, China

Mr. C. Liu
State Key Laboratory of Structural Analysis for Industrial Equipment, Dalian University of Technology, Dalian, 116024, China

Dr. S. Bi
Key Laboratory for Precision and Non-traditional Machining, Technology of the Ministry of Education, Dalian University of Technology, Dalian 116024, China

Dr. Y. Liu, Mr. Q. Liu, Dr. M. Lu, Prof. Z. Jing, Prof. W. Peng
School of Physics, Dalian University of Technology, Dalian 116024 China
Corresponding author e-mail: wpeng@dlut.edu.cn and liuyun89@dlut.edu.cn

1. Near-infrared (NIR) Surface Plasmon Resonance (SPR) Sensing

1.1 Drude model parameters

As shown in **Table S1**, due to $\Omega_p = 2\pi c/\lambda_p = \sqrt{f}\omega_p$, we can obtain plasma wavelength ($\lambda_p = 1.576 \times 10^{-7}$m).

Table S1 Values of the Drude model parameters for the permittivity of gold.

Material	ω_p	f	γ
Au	1.372×10^{16}	0.760	0.053

1.2 Properties of NIR-SPR with different thicknesses

NIR-SPR with different thicknesses of gold metal film possess different characteristics of SPR reflection spectra. Based on the resonance effects of surface plasma, there are two resonance conditions: (i) the incident light is p-polarized light; (ii) the wave vector and

frequency of incident light wave and surface plasma wave must be consistent. Thus, here we calculate the reflection coefficient of p-polarized light. The Kretschmann's structure consists of prism, metal film and sample. Combined with membrane theory and Fresnel formula, the reflection coefficient of p-polarized light on the prism can be expressed as:

$$R^p = \left|r_p\right|^2 = \left|\frac{r_{p(0,1)}+r_{p(1,2)}e^{2i\phi}}{1+r_{p(0,1)}r_{p(1,2)}e^{2i\phi}}\right|^2 \quad \text{(S1)}$$

$$r_{p(k,k+1)} = \frac{n_{k+1}cos\theta_k - n_k cos\theta_{k+1}}{n_{k+1}cos\theta_k + n_k cos\theta_{k+1}} \quad (k = 0, 1) \quad \text{(S2)}$$

$$\theta_k = arcsin\frac{n_0 sin\theta_0}{n_k} \quad (k = 1, 2) \quad \text{(S3)}$$

$$\phi = \frac{2\pi d n_1 cos\theta}{\lambda} \quad \text{(S4)}$$

where $r_{p(k,k+1)}$ is the reflection coefficient of incident light at the interface between two adjacent layers of media, n_k is the refractive index (RI) of the k-layer medium and d is the thickness of the metal film. Next, the reflection spectra of NIR-SPR with different thicknesses of gold metal film were simulated as shown in **Figure S1**a. And the experimental spectra were also recorded as shown in Figure S1b. It can be found that the reflection spectra of NIR-SPR with different thicknesses of gold metal film are different, both in simulation and in experiment. In order to study the characteristic of simulation and experimental spectra, several spectral properties were analyzed. As shown in Figure S1c-d, we can find that resonance depth and half peak width of spectra decrease with the increasing thickness of gold film. The experimental results give good agreement with simulation result. We further researched the sensitivity and figure of merit (FOM), as shown in Figure S1e-f. As the thickness of gold film increases, sensitivity and FOM increase firstly and then decrease. FOM is an important parameter of SPR, which can examine the ability to sensitively measure very small signal changes. FOM processes the maximum value when the thickness of gold film is 50 nm in simulation and experimental results. We chose 50 nm as the thickness of gold film in our experiment.

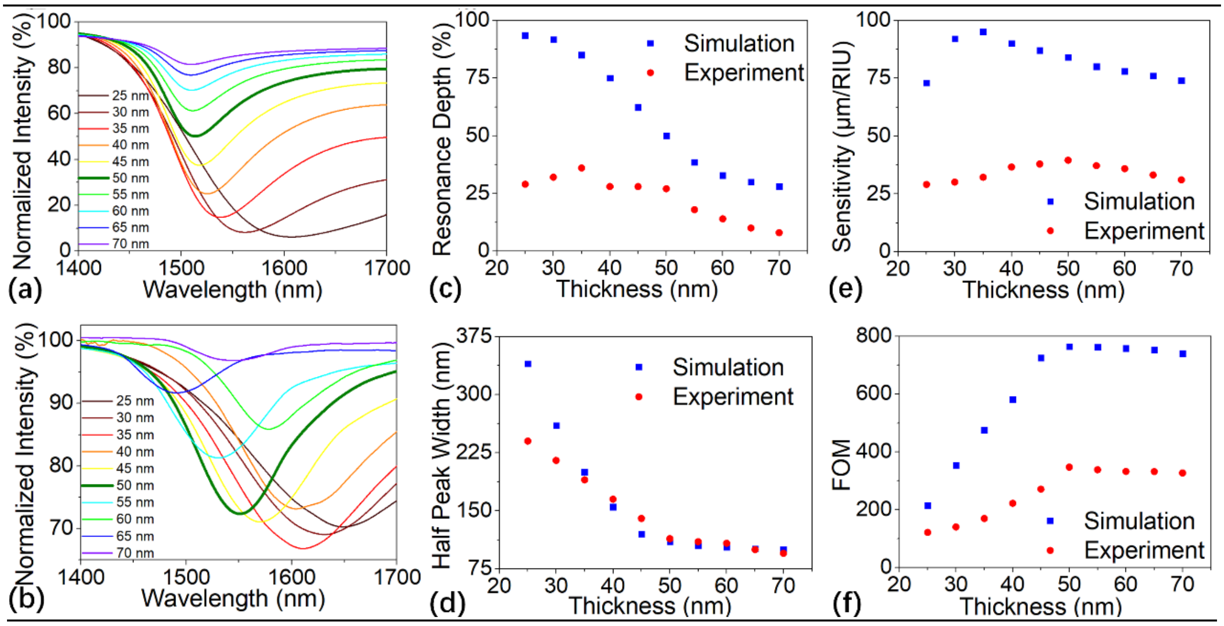

Figure S1 Characteristics of NIR SPR sensor with different thickness of gold film. (a) reflection spectra of simulation (a) and experimental result (b), (c) Resonance depth (c), half peak width (d) Sensitivity (e) and FOM (f) of simulation and experimental result

1.3 Electric field intensity distributions

In order to further research the SPR field distribution characteristics of different resonant wavelengths, the electric field distributions (x component of the field Ex, y component of the field Ey and intensity |E|) for visible and NIR bands were simulated by COMSOL software as shown in **Figure S2**a-c. TM waves are directed along 5° and 28° from x-axis at wavelengths of 570 nm and 1550 nm. The gold-water interface exhibits a prominent evanescent field in the surrounding medium. It can be found in Figure S2d that the intensity and depth of decay field in NIR band is much greater than that in the visible band. The improvement in the intensity and depth of decay field inside the dielectric can lead to the sensitivity enhancement at long wavelength bands, because decay field of SPW around sensing surface is sensitive to RI variation of the dielectric medium.

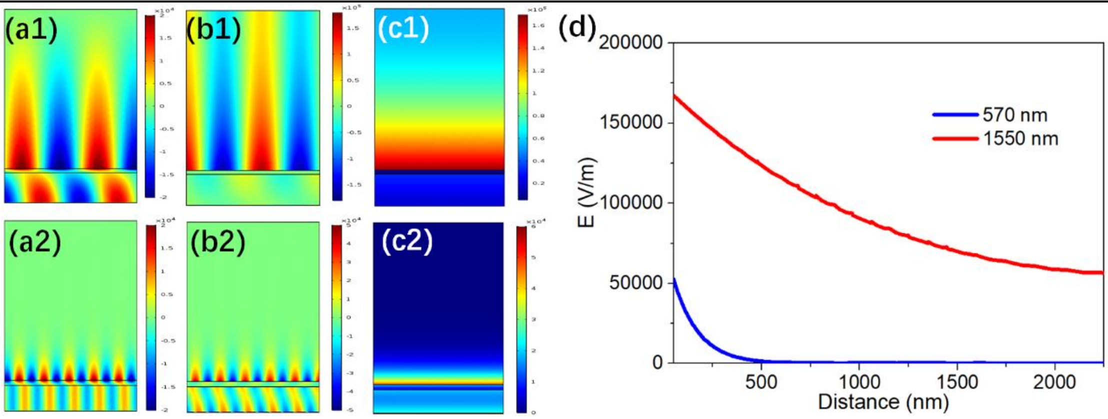

Figure S2 Electric field distribution. X component of the field Ex (a1-2), Y component of the field Ey (b1-2) and electric field intensity |E| (c1-2) appearing in NIR band (1550 nm, label 1) and the visible band (570 nm, label 2) and NIR band (1550 nm, label 1). (d) Electric field intensity distribution of SPR in NIR and visible band.

1.4 Decay depth of surface plasmon

The real part of the gold RI decreases significantly at longer wavelengths, as shown in **Figure S3**a. The operating wavelength is set as 400-2000 nm. The decay length inside the metal is affected by the permittivity of the metal film and expressed as

$$\delta_d = \frac{\lambda}{2\pi}\sqrt{\frac{\varepsilon_d+\varepsilon_{mr}}{-\varepsilon_d^2}} \tag{S5}$$

The decay length (δ_d) is a function of the real part of permittivity (ε_{mr}). We set the water with a RI of 1.33 as the dielectric medium. The decay length can be calculated and plotted in Figure S3b. It indicates that there is the greater decay length of SPW associated with surface plasmons at the longer wavelength band, which corresponds to the analysis of sensitivity above.

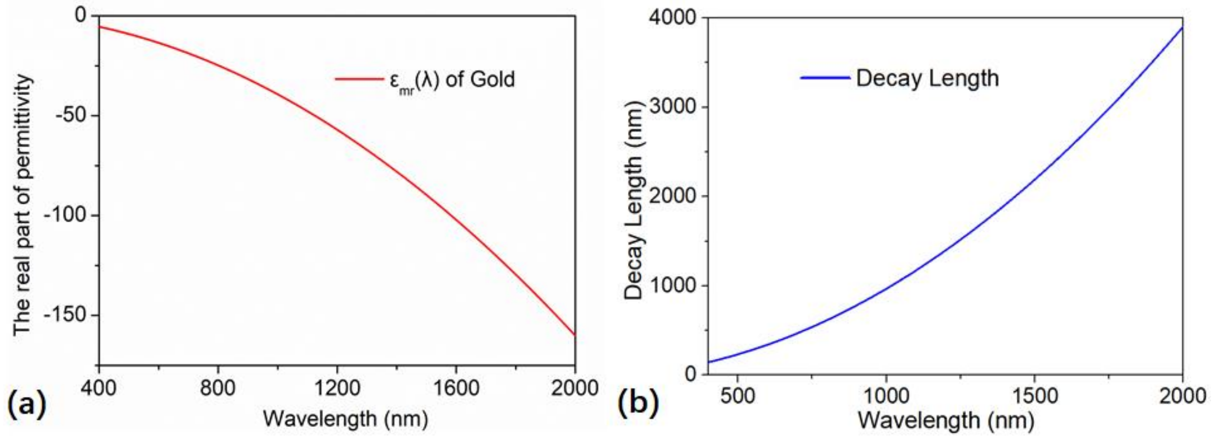

Figure S3 Theoretical analysis of SPR sensor with gold sensing film, the operating wavelength is set as 400-2000 nm. (a) Real part of permittivity of the gold calculated using Drude model (b) Decay length inside the metal with different wavelengths, where the water with a RI of 1.33 is set as the dielectric medium.

1.5 Relationship between Ris and reflection spectra with different incident angles

The reflection spectra with different incident angles are calculated, and spectral response with different RIs are shown in **Figure S4**.

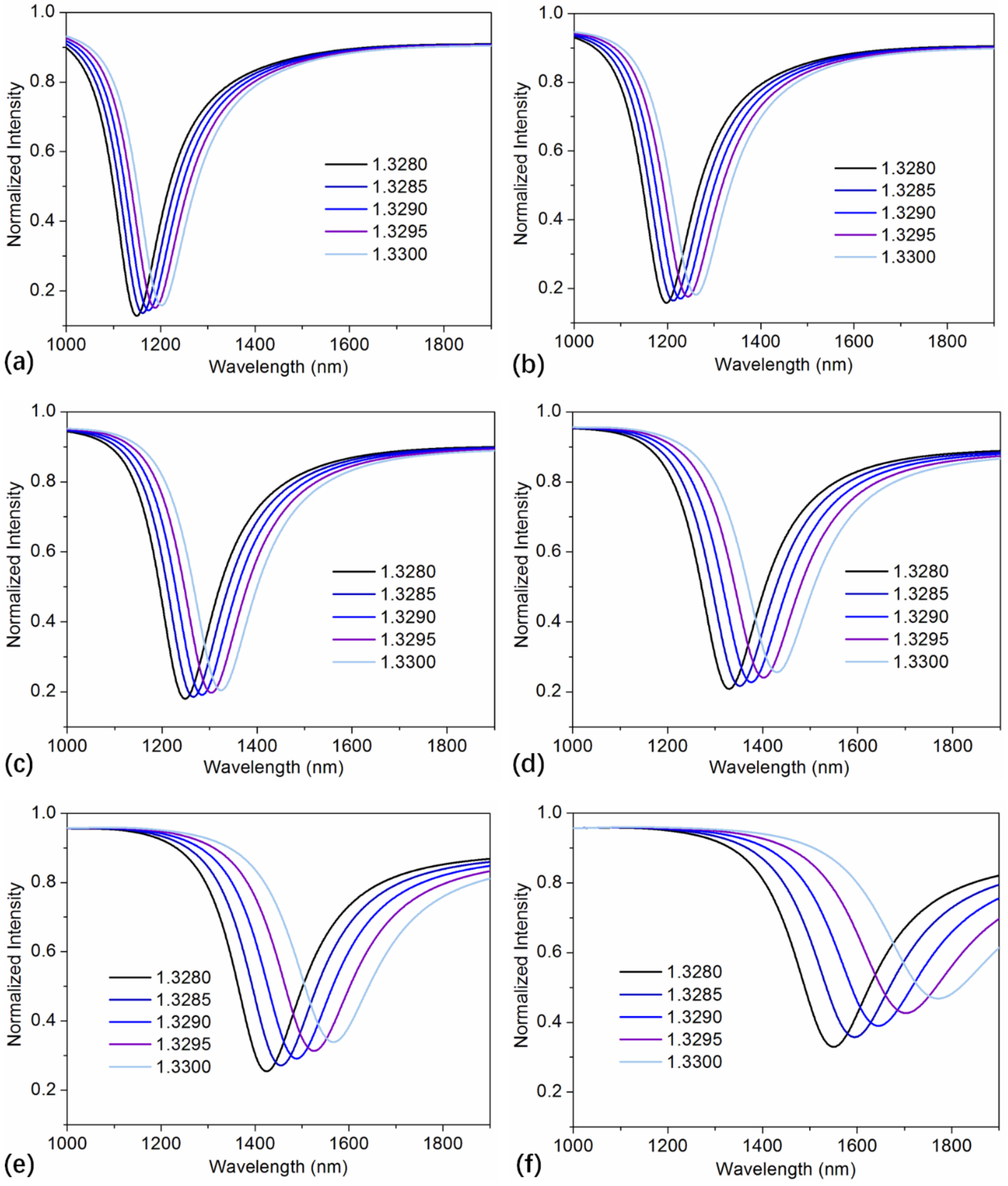

Figure S4 Reflection spectra simulation result of NIR-SPR sensing elements for different RIs with incident angles of 63.06°, 62.90°, 62.76°, 62.58°, 62.42° and 62.27°.

2. Surface-Sensitivity Enhancement using the Gold-coated nanotriangular (NT) Array

2.1 COMSOL modeling of one complete period

A simulation model structure of one complete period was built, as shown in **Figure S5**.

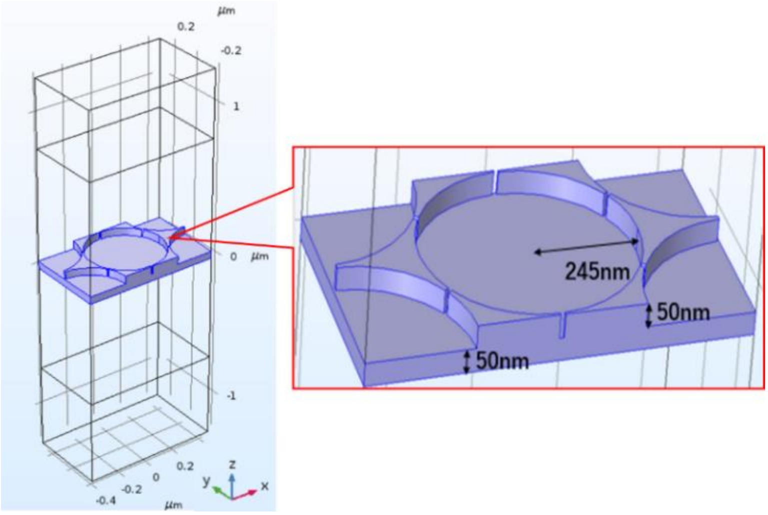

Figure S5 NT model structure of one complete period by using a COMSOL simulation.

2.2 Simulation result of different dimension of gap

The dimension of gap includes the height of gap and the distance between both tips of adjacent NT plates. In order to elaborate the property of sensing element with different dimension of gap, we simulated the characteristic of spectrum and electric field. First, we selected 300 nm, 500 nm and 700 nm as the diameters of PS spheres, and the corresponding distance between both tips of adjacent NT plates are approximately 38 nm, 50 nm and 60 nm (the height of gap is set as 50 nm). As shown in **Figure S6**a-c(1), it can be found that the resonance wavelength is red-shifted with the diameter decreasing while spectral shapes are similar. Then, we selected 40 nm, 50 nm and 60 nm as the heights of gaps (the diameters of PS sphere and gap size are set as 500 nm and 50 nm). As shown in Figure S6a1 and Figure S6 d-e(1), the resonance wavelengths of similar spectral shapes are red-shifted when the gap height increases. The resonance wavelengths of different gap sizes can be adjusted by incident angle. And the above dimensions of gap can be chosen based on analysis of spectral

characteristics. Second, electric field distributions of the above dimensions over a single period in X-Y plane and cross section are numerically evaluated using the same color code (all resonance wavelengths are adjusted to 1550 nm and selected as the incident light wavelength) as shown in Figure S6a-e(2-3). For different dimensions of gap, the central areas of the NT terraces all exhibit larger field intensity distributions than the gold film. The local field distributions of NT array show similar property that there are strong enhancements around edge and vertex. The local field strength in the vicinity of a sharp tip can be enhanced by several orders of magnitude compared with the incident light. We can find that overall strength of electric fields for different dimensions of gap are different, which is due to the different absorbances under resonance wavelengths. However, metallic nanostructures with sharp corners or edges are especially favorable for the detection of specific interactions between biologically relevant species. And the enhancement of local field is more important than overall field strength.

Based on the above analysis, the NT array with the above dimensions shows similar performance. Furthermore, the sensing structures also provide coupling enhancement effect with gold nanoparticles (GNPs) around gap. Considering the size of GNP (40 nm) we used in our experiment, GNP can be better coupled to both tips of adjacent NT plates at the same time when 50 nm is set as the distance between both tips of adjacent NT plates. Changes in height have little effect on coupling because GNPs are randomly bound to the sensing surface via DNA. Thus, we chose 50 nm as the height and 500 nm as the diameter of the PS sphere. At this time, the corresponding gap size is approximatively 50 nm.

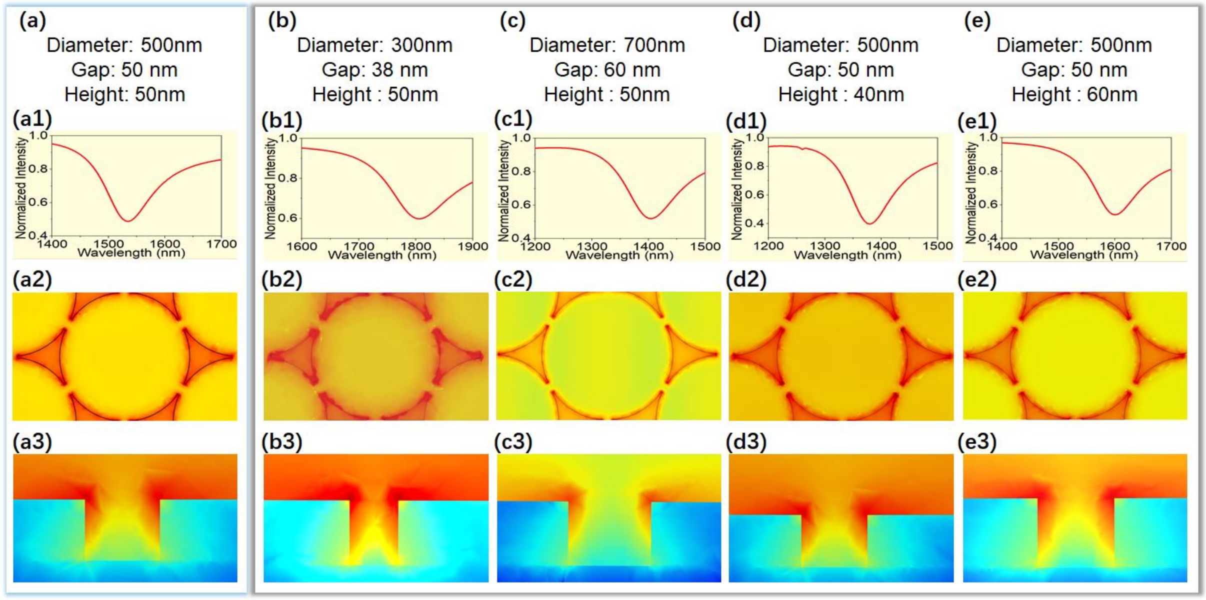

Figure S6 Characteristic of spectrum and electric field of sensing element with different dimension of gap

2.3 Comparison of flat gold film and continuous gold-coated NT array

Simulation results for spectra of NIR-SPR sensor based on flat gold film and gold-coated NT array at different incident angles are shown in **Figure S7**a-b. For two cases, the resonance wavelengths increase as the incident angles decrease. Resonance wavelengths are very sensitive to incident angles. The change by 1° can cause the wavelength red-shift of more than 100 nm. The introduction of NT array only affects the values of resonance wavelengths, and that can be adjusted by decreasing the incident angles. Simulation results of electric field distribution (x component of the field E_x) corresponding to flat gold film and gold-coated NT array are shown in Figure S7c-d. The electric field distributions of a large region in dielectric layer are similar for two cases, and the introduction of NT array only causes enhancement of the near field around the array.

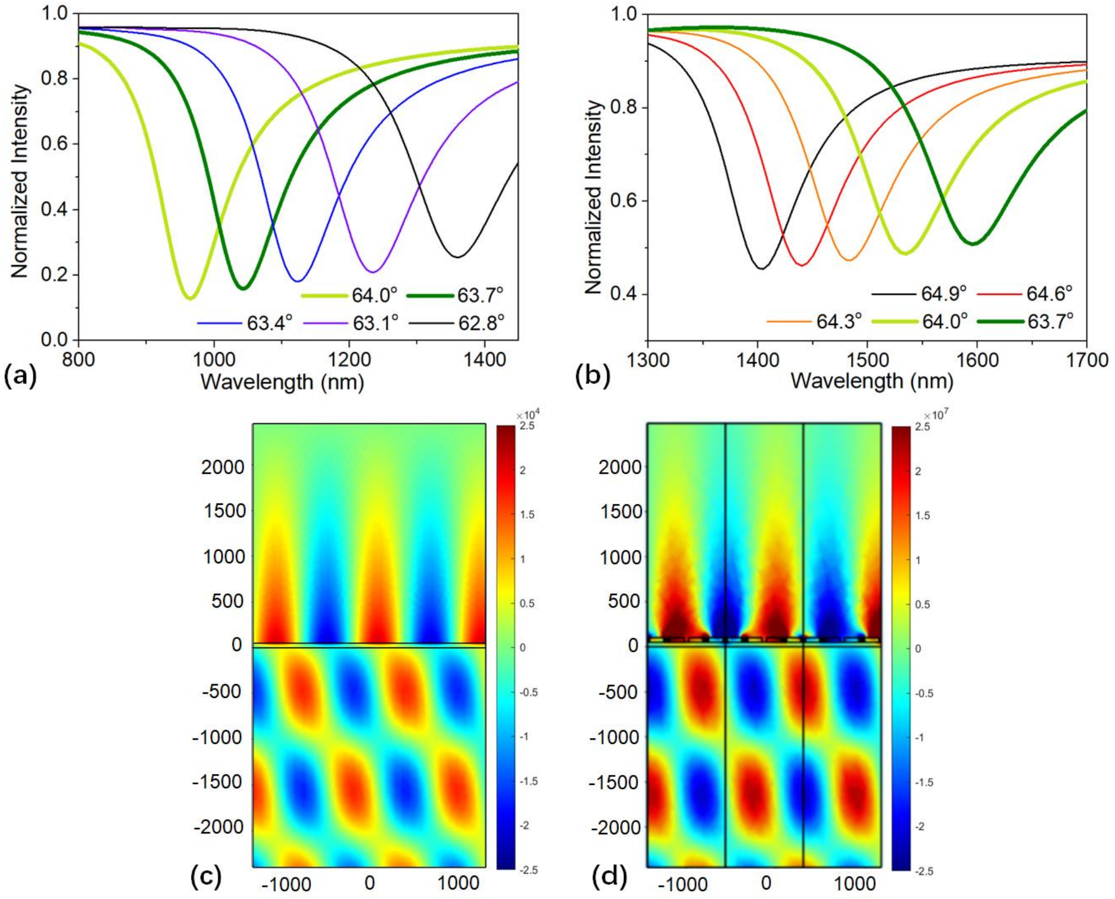

Figure S7 Simulation results of spectra of NIR-SPR sensor based on flat gold film (a) and gold-coated NT array (b) with different incident angles. Simulation results of electric field distribution (x component of the field E_x) corresponding to flat gold film (c) and gold-coated NT array (d)

2.4 Experimental errors for the resonant wavelength

Here, we researched the experimental errors for the resonant wavelength. We recorded 20 groups of SPR reflection spectra by using our experimental platform, as shown in **Figure S8**a-b. Then, resonant wavelengths were identified by spline interpolation algorithm. The resonant wavelengths were recorded as shown in Figure S8c-d. During the experiment, we tried our best to ensure that all experimental conditions remained unchanged, but some noise signals still existed. The experimental errors for the resonant wavelengths of two sensing chips are ±28.5 pm and ±43.0 pm, respectively. Therefore, the experimental error of spline interpolation algorithm for the resonant wavelength is less than ±28.5 pm.

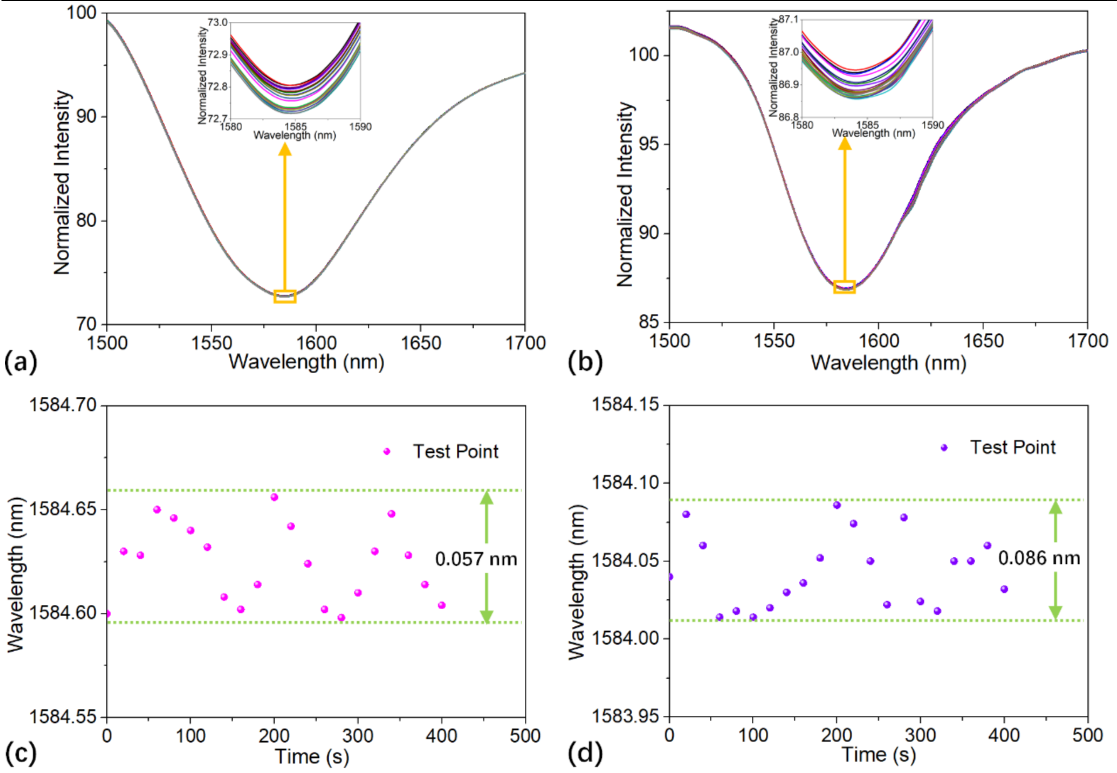

Figure S8 (a-b) Recorded transmission spectra of two sensing chips in 400 s; (c-d) Wavelength shift of resonance peak corresponded in (a-b)

3. Biosensing application of NIR nanoplasmonic sensor

3.1 Enhancement of near field intensity by introducing NT array

Due to the introduction of NT array, the sensing element possesses higher surface-sensitivity and can measure directly ssDNA hybridization. The introduction of NT array causes enhancement of the near field around the array, which is clearly demonstrated in **Figure S9**a-b. The magnified views of near field intensity are shown in Figure S9c-d. For NT array, there are three structural cases, including the plane, edge and vertex. Compared with flat gold film, the near-field distribution shows the strong enhancement around edge and vertex. For all cases of NT array, vertex resulted in the strongest electric field, followed by the edge, while the plane resulted in the one that is similar to flat gold film. The strong enhancement effect of near-field can result in high surface-sensitivity.

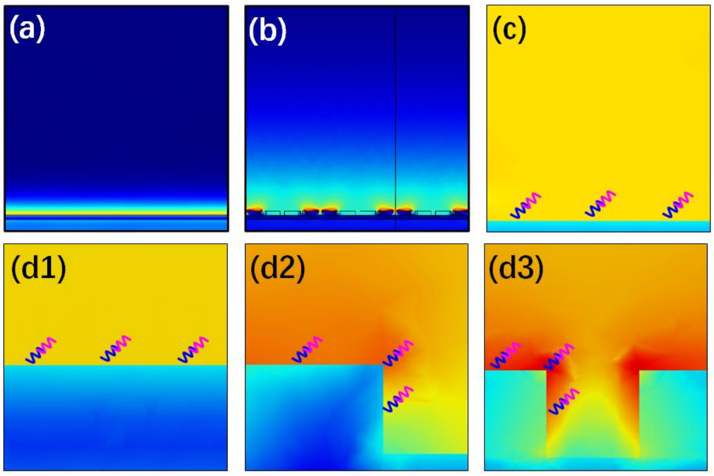

Figure S9 Distribution of electric field intensity (|E|) corresponding to flat gold film (a) and gold-coated NT array (b). Near-field distribution corresponding to flat gold film (c) and gold-coated NT array (d1-4).

3.2 Comparison of electric field with/without GNPs

In order to better compare the electric field changes when GNPs are introduced to carry out coupling with the NT plates, we studied the electric field distributions in both directions: (i) X-Y plane and (ii) the different planes perpendicular to the sensing surface.

(i) Electric field distributions numerically evaluated over a single period in the X-Y plane for Z=65 nm (the plane through the center of GNPs) and Z=50 nm (the bottom gold film surface) are shown in **Figure S10**a-b and Figure S10c-d, respectively. By comparing Figure S10a(1-2) (with GNPs) with (b1-2) (without GNPs), there are obvious coupling effects of GNPs with the vertex and side surface of NT plate. The stronger electric coupling occurs when the GNP is attached to the vertex. By comparing Figure S10c(1-2) (with GNPs) and (d1-2) (without GNPs), there are obvious coupling effects of GNPs with the bottom surface. We can find that the intensities of near-field between GNPs and gold surface are enhanced after the addition of GNPs.

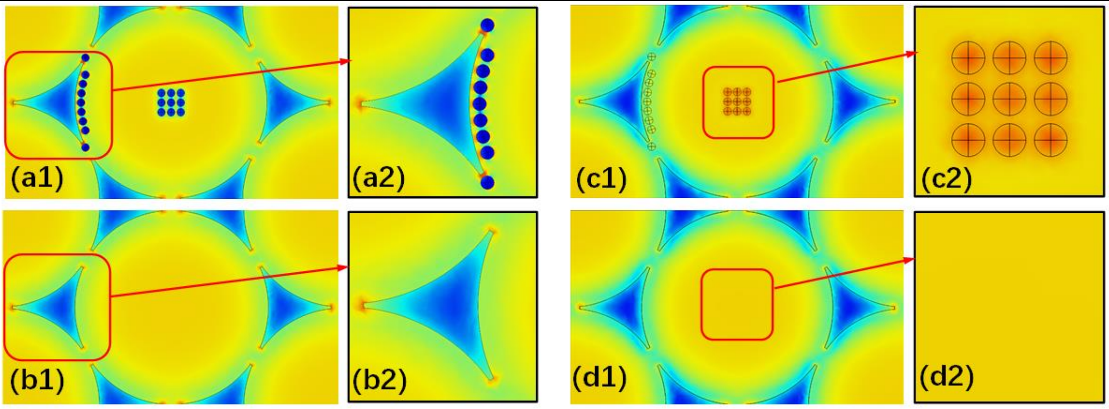

Figure S10 Electric field distributions numerically evaluated over a single period in the X-Y plane for Z=65 nm (a-b) and Z=50 nm (c-d)

(ii) The electric field distributions are evaluated numerically in different planes perpendicular to the sensing surface. By comparing label 1 (with GNPs) with label 2 (without GNPs) in each group, it can be found that GNPs can exhibit the asymmetric field distribution, and the electric fields are concentrated in the gap between the GNPs and the gold film. This result indicates that there is a strong coupling after the addition of GNPs. **Figure S11** shows that the electric field can be enhanced by GNPs at different locations. Of all cases considered, the GNP located at the vertex resulted in the strongest coupling, followed by the GNP located at the edge, while the GNP located on the plane resulted in the weakest coupling.

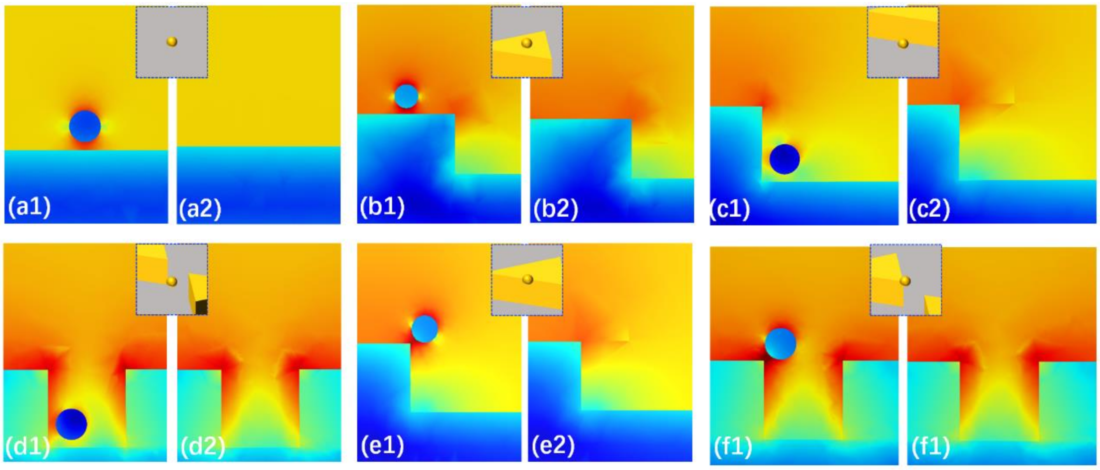

Figure S11 Simulations of the electric field distributions with/without GNPs at different locations of an NT array: GNP is placed near (a) the single gold film, (b) the terrace of the NT plate, (c) the side face of the NT plate, (d) the lateral edge of the NT plate, (e) the edge of the terrace of the NT plate, and (f) the vertex of the NT plate.

3.3 Comparison between GNPs with different diameters

To demonstrate the enhancement effect, we detected the 100 nM *RpoB* solution by using GNPs with diameters of 20 nm and 40 nm. Clearly, the employment of the signal amplifiers greatly enhanced the binding signal, as shown in **Figure S12**a. Next, we researched the size dependence of GNPs-enhanced plasmonic sensor. The enhancement effect increases first and then decreases with the increasing size of GNPs. This is related to the coupling, extinction and scattering effect of GNPs, as well as the affinity and steric hindrance of GNPs. [1-4] The optimal value is typically between 40 nm and 100 nm, and there are certain differences for different sensing elements. In our experiments, GNPs with a diameter of 40 nm can obtain the larger enhancement than the GNPs with a diameter of 20 nm. To further prove our experimental result that 40nm nanoparticles have better enhancement effect, we simulated two cases of different diameters of GNPs. It is easy to find from Figure S12b-g that there are strong dipolar modes excited between the NT plate and the GNP with different sizes. The electric field distributions of GNPs with different sizes are similar at the same location. In the gap between the GNP and the sensing surface, near fields are stronger and wider for the GNPs with larger size. In addition to coupling effect, the large permittivity and surface mass loading of GNPs also enhance the surface-sensitivity of plasmonic sensor. The GNP with a diameter of 40 nm possesses greater permittivity and surface mass loading, which leads to greater surface-sensitivity.

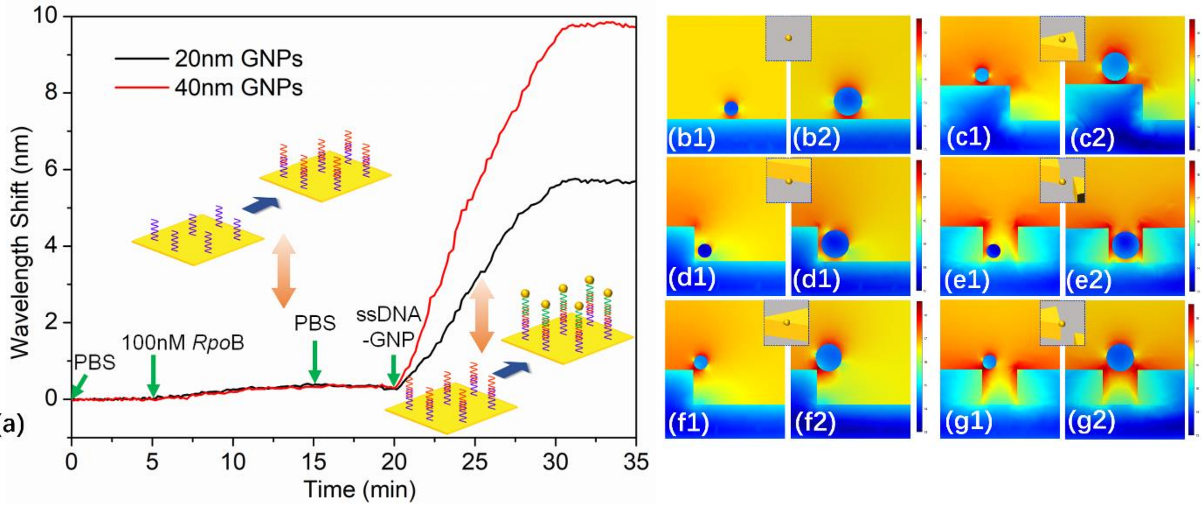

Figure S12 (a)Wavelength shift of the sensor for detecting 100 nM *RpoB* by using GNPs with diameters of 20 nm and 40 nm. Simulations of the electric field distributions for the coupling between a single GNP at different locations of an NT array (label 1 and 2 correspond to GNPs with 20nm and 40nm diameters, respectively): The GNP is placed near (b) the single gold film, (c) the terrace of the NT plate, (d) the side face of the NT plate, (e) the lateral edge of the NT plate, (f) the edge of the terrace of the NT plate, and (g) the vertex of the NT plate.

3.4 The wavelength response of the sampe with the concentration below detection range

We detected RpoB solution at concentrations of 10^{-19}-10^{-18} M by sandwich amplification strategy. **Figure S13** shows the real-time responses, the wavelength red shifts due to *Rpo*B binding to capture ssDNA and surface adhesion influence in the sensing region. In our paper, the LOD is defined as the smallest distinguishable sample concentration, which is equal to the sum of the blank signal and three times the standard deviation of the signal noise level. It can be found that the response differences between samples with and without *Rpo*B are less than 0.06 (triple standard deviation). The response signal of *Rpo*B solution at concentrations below 10^{-18} M is difficult to be accurately distinguished from the signal of surface adhesion influence, so they cannot be used as the effective experiment data.

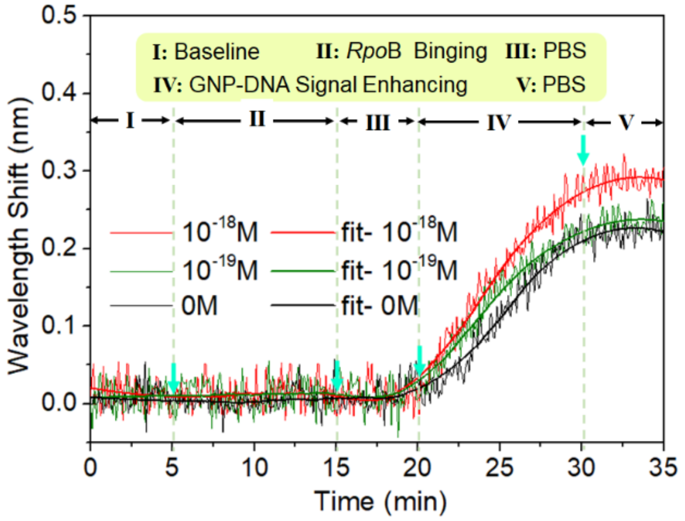

Figure S13 Wavelength responses of RpoB hybridization by using GNP amplification tags at concentrations of 0 M, 10^{-18} and 10^{-19} M.

3.5 Hybridization conditions of the single nucleotide mismatch ssDNA

Single nucleotide mismatch ssDNA and noncomplementary ssDNA (KatG) at concentrations of 10^{-7} M under the same condition were analyzed as shown in **Figure S14**. The single nucleotide mismatch ssDNA may bind with the capture ssDNA and GNP-DNA under the following conditions: (a) hybridization occurs over only half of the sequence; (b) complete hybridization occurs with a small bulge in the mismatch area; (c) cross-hybridization occurs between two neighboring ssDNA, which results in weaker binding and more space than hybridization of *RpoB*.

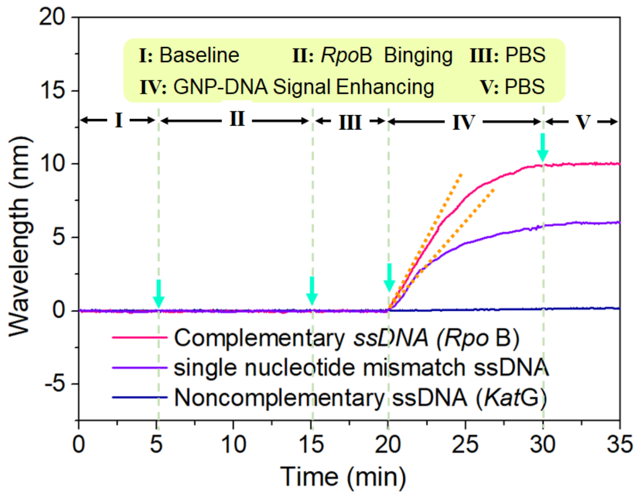

Figure S14 Wavelength shift in the concentration 10^{-7} M for hybridization of GNP-DNA and RpoB/single nucleotide mismatch ssDNA/KatG.

3.6 Reproducibility tests of ssDNA hybridization

In order to verify the reproducibility of our experimental results, the sensing chip was regenerated in high-concentration urea (8M) by ultrasonication at high temperature (80°) for 60 min. First, we tested the *Rpo*B at a concentration of 10^{-7} M in three replicated tests, as shown in **Figure S15**a. We can find that our sensor possesses good reproducibility and the wavelength shifts of three tests are similar. Then, each measurement at a given concentration was repeated thrice. Wavelength resonances during the hybridization of GNP-DNA and *Rpo*B are shown in Figure S15b. The error bars in the diagram indicate the relative errors of the variability among samples at the same concentration in three replicated tests. The standard deviations of all reproducibility tests were less than 0.629. Furthermore, the experimental results of replicated tests for *Kat*G and mismatch ssDNA were also shown in **Table S2**.

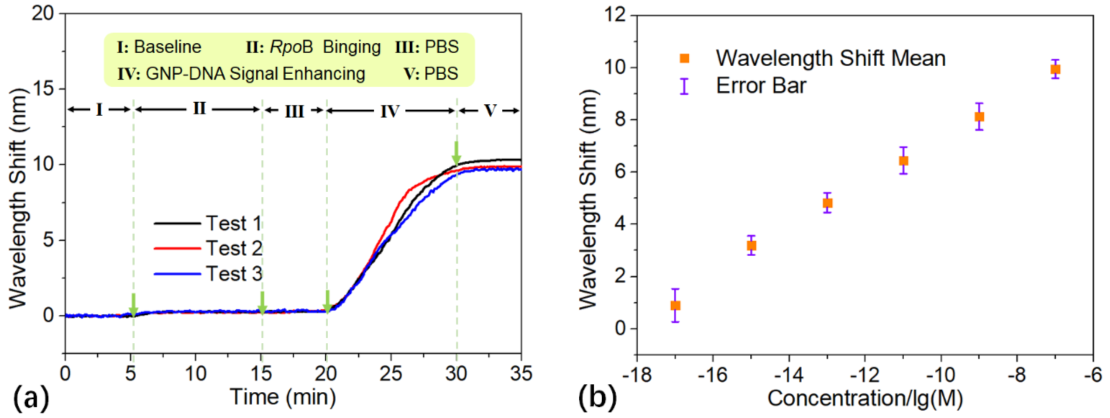

Figure S15 Wavelength shift (a) and resonance (b) during the hybridization of GNP-DNA and *Rpo*B in three replicated tests

Table S2 Experiment results of reproducibility tests.

Concentration	*Rpo*B		Single nucleotide mismatch ssDNA		*Kat*G	
	Mean	Standard deviation	Mean	Standard deviation	Mean	Standard deviation
10^{-7} M	9.944	0.354	6.665	0.393	0.271	0.141
10^{-9} M	8.128	0.510	5.417	0.398	0.278	0.156
10^{-11} M	6.446	0.506	4.202	0.492	0.277	0.120
10^{-13} M	4.831	0.376	3.135	0.438	0.248	0.141
10^{-15} M	3.200	0.359	2.078	0.386	0.278	0.113
10^{-17} M	0.902	0.629	0.448	0.218	0.271	0.127

3.7 Surface RI sensitivity calculation

Here, we discuss the surface-sensitivity in terms of RI. Plasmonic sensing is ideally suited to detect surface bioaffinity adsorption of various biological and chemical analytes. The value of surface concentration (M) of molecular is calculated by the following formula,[5, 6]

$$M = \frac{d_p(n_p - n_b)}{dn/dc} \tag{S6}$$

Where d_p is the effective thicknesses of molecular layer, n_p is the effective RI, n_b is the buffer RI, $dn/dc = 0.182$ mL/mg[6] and $\Delta n = n_p - n_b$ is RI variation caused by the binding of molecules to the sensing surface. In our work, d_p is the thickness of ssDNA, which is approximately 3 nm, and the molecular weight (mw) of the target ssDNA is

$7.3\ kDa$, i.e. $7.3 \times 10^3\ g/mol$. Based on the LOD 1.2 aM in our work, the limit of concentration of ssDNA (C_{min}) can be expressed that

$$C_{min} = LOD \times mw = 8.76 \times 10^{-15}\ g/L \tag{S7}$$

In our work, binding saturation was reached when 1ml ssDNA solution (V) was pumped. Therefore, the minimum mass of ssDNA (m), which can be detected within the range of the sensing chip, can be expressed that

$$m = C_{min} \times V = 8.76 \times 10^{-18}\ g \tag{S8}$$

If the sensing area is set as A, we can obtain that

$$M = \frac{m}{A} = 2.19 \times 10^{-18}\ g/\ cm^2 \tag{S9}$$

Based on Equation (6), RI variation (Δn) can be calculated as 1.3286×10^{-9} RIU. The surface RI sensitivity can be obtained that

$$S = \frac{\Delta\lambda}{\Delta n} = 4.742 \times 10^7\ nm/RIU \tag{S10}$$

The surface RI sensitivity of the above calculation is only an estimate based on the condition that all molecules pumped into flow cell are bound to the sensing surface, but there are some molecules that are not bound to the sensing surface in the experiment. Hence, the real surface RI sensitivity is more than $4.742 \times 10^7\ nm/RIU$. We can find that our surface RI sensitivity is much larger than the bulk RI sensitivity due to the local field enhancement around NT array and GNPs coupling.

4. Experimental Section

4.1 Sequences of the oligonucleotides used

All DNA single strand sequences are listed in **Table S3**. All ssDNA were purified by HPLC_CE(IVD) method.

Table S3 Sequences of the oligonucleotides used.

Oligonucleotide	Sequence (5'-3')
**Capture ssDNA**	/3ThioMC3-D/AG GGG CCC AAC
**Probe ssDNA**	ATC GGT CTG ATA AAA AA/3ThioMC3-D/
**Target ssDNA (*RpoB*)**	ATC AGA CCG ATG TTG GGC CCC T
**Single nucleotide mismatch ssDNA**	ATC AGA CCG ATG TTG GGC ACC T
**Noncomplementary ssDNA (*Kat*G)**	TTG ACC TCC CAC CCG ACT TGT G

4.2 Experimental set-up

Figure S16 is the experimental set-up of our sensing system. Our sensing chip is a glass sheet and continuous gold-coated NT array was fabricated throughout the total glass sheet (20 mm×20 mm×1 mm). The flow cell (Φ15 mm×2 mm) was customized and fixed in to sensing area. The spot of the broadband light source (Φ5 mm) is irradiated to the sample flow area and located in the center position.

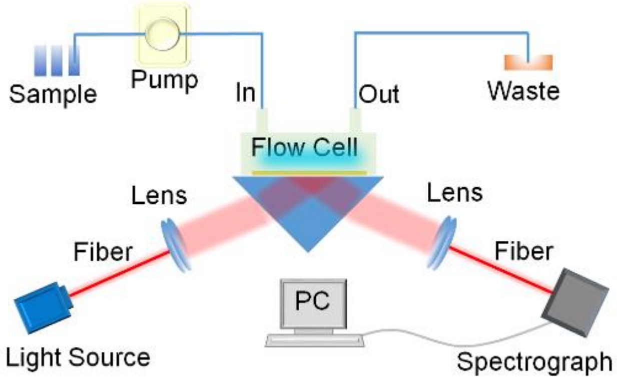

Figure S16 Experimental Set-up of the sensing system.

4.3 NT array sensing structure fabrication

First, the PS spheres self-assembled into a large-area HCP pattern, and a 50 nm gold film was deposited onto the substrate with HCP monolarer PS spheres by thermal evaporation. PS spheres were removed and a 50 nm gold film was coated on the nanostructured film. By this preparation process in **Figure S17**, continuous gold-coated nanotriangular array was fabricated.

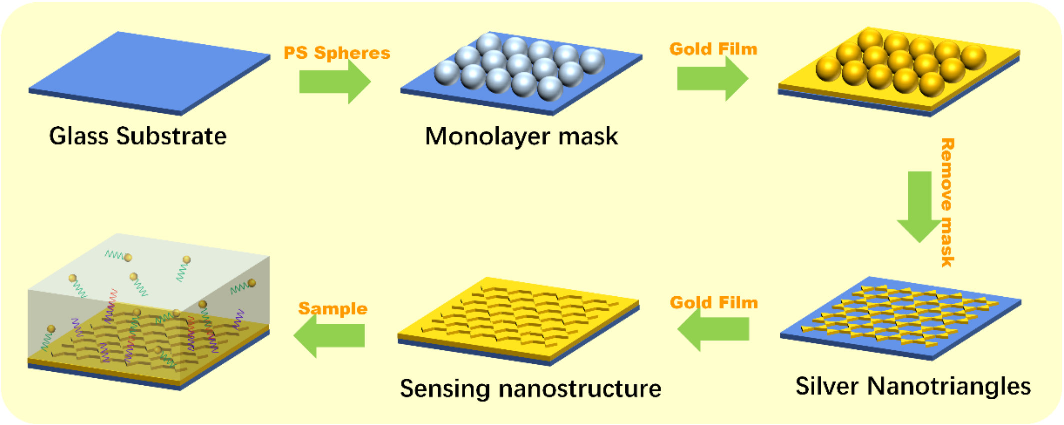

Figure S17 Fabrication processing of the gold-coated NT sensing structure.

4.4 Preparation of GNP Solutions

GNP solutions with diameters of 20 nm and 40 nm were produced by using the citrate reduction method and observed in TEM image in **Figure S18**.

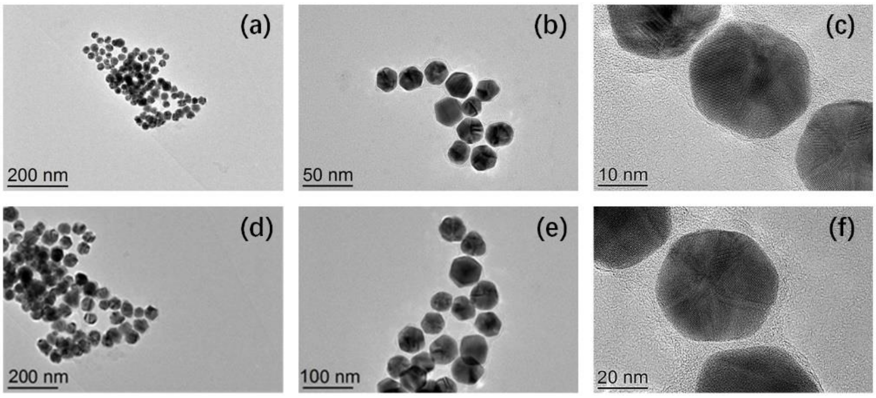

Figure S18 TEM images of GNPs with diameters of 20 nm (a-c) and 40 nm (d-f).

4.5 Functionalization of GNP solutions with ssDNA

Successful incorporation of ssDNA onto the GNP was verified using a method developed by Mirkin teams.[7-9] The absorbance experienced a red shift of wavelength and significant decrease in intensity when GNPs were assembled by linking DNA into extended structures, which also resulted in a color change of the solution. It was also proved in our work (see **Figure S19**). This is due to the formation of nanoparticle aggregates. The plasmon

band as characteristic of GNPs is very sensitive to interparticle distance as well as aggregate size. The observation of absorbance can provide compelling evidence to verify the synthesis of GNP/DNA complex. This method was reported in ref. [8] and exemplified by experimental research on the reversible formation and dissociation of GNP-DNA aggregates. In recent years, this method was also widely used to detect the synthesis of DNA and GNP in many reports.[10-13]

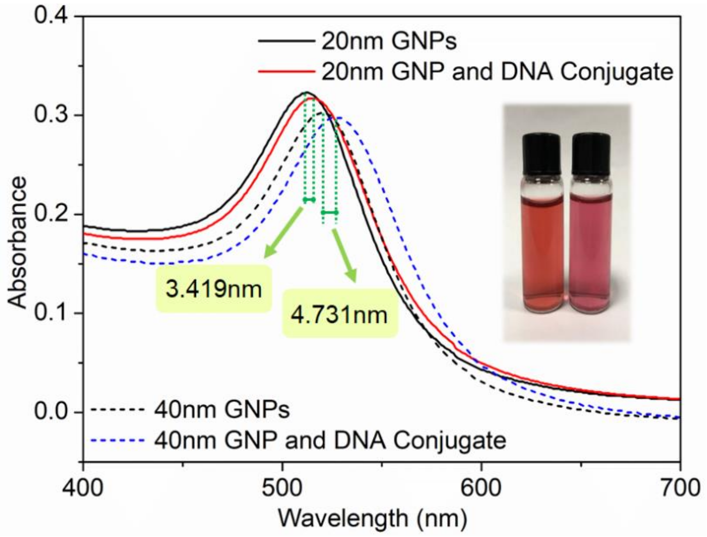

Figure S19 UV-vis spectra of the GNP solution, including GNPs with diameters of 20 nm (black solid line) and 40 nm (black dashed line) and DNA-functionalized GNPs with diameters of 20 nm (red solid line) and 40 nm (blue dashed line). The inset: Photograph of the color change from red to pinkish after DNA functionalization

References

[1] J. Li, H. Duan, P. Xu, X. Huang, Y. Xiong, *Rsc Adv*. **2016**, 6, 26178.

[2] C. T. Yang, Y. Xu, M. Pourhassan-Moghaddam, D. P. Tran, L. Wu, X. Zhou, B. Thierry, *Sensors*. **2019**, 19, 323.

[3] L. A. Lyon, D. J. Pena, M. J. Natan, *J. Phys. Chem. B.* **1999**, 103, 5826.

[4] L. He, M. D. Musick, S. R. Nicewarner, F. G. Salinas, S. J. Benkovic, M. J. Natan, C. D. Keating, *J. Am. Chem. Soc.* **2000**, 122, 9071.

[5] J. W. Corsel, G. M. Willems, J. M. M. Kop, P. A. Cuypers, W. T. Hermens, *J. Colloid. Interf. Sci*. **1986**, 111, 544.

[6] V. Silin, H. Weetall, D. J. Vanderah, *J. Colloid. Interf. Sci*. **1997**, 185, 94.

[7] R. Elghanian, J. J. Storhoff, R. C. Mucic, R. L. Letsinger, C. A. Mirkin, *Science*. **1997**, 277, 1078.

[8] C. A. Mirkin, R. L. Letsinger, R. C. Mucic, J. J. Storhoff, *Nature*. **1996**, 382, 607.

[9] J. J. Storhoff, C. A. Mirkin, *Chem. Rev*. **1999**, 99, 1849.

[10] Q. Wang, Q. Li, X. Yang, K. Wang, S. Du, H. Zhang, Y. Nie, *Biosens. Bioelectron*. **2016**, 77, 1001.

[11] M. Lu, L. Hong, Y. Liang, B. Charron, H. Zhu, W. Peng, J. F. Masson, *Anal. Chem*. **2018**, 90, 6683.

[12] Q. Wu, Y. Sun, P. Ma, D. Zhang, S. Li, X. Wang, D. Song, *Anal. Chim. Acta*. **2016**, 913, 137.

[13] K. Li, G. Liu, Y. Wu, P. Hao, W. Zhou, Z. Zhang, *Talanta*. **2014**, 120, 419.
